# Supplementary material for: Broad-spectrum chemicals block ROS detoxification to prevent plant fungal invasion
Source: Curr Biol. Author manuscript; Available in PMC 2022 Oct 13. (PMC7613639; doi:10.1016/j.cub.2022.07.022)
Supplement: Supplementary Information [file EMS152247-supplement-Supplementary_Information.zip › 1-s2.0-S096098222201123X-mmc1.pdf]

Current Biology, Volume *32*

Supplemental Information

Broad-spectrum chemicals block ROS detoxification to prevent plant fungal invasion

Qianqian Yang, Jinguang Yang, Yameng Wang, Juan Du, Jianan Zhang, Ben F. Luisi, and Wenxing Liang

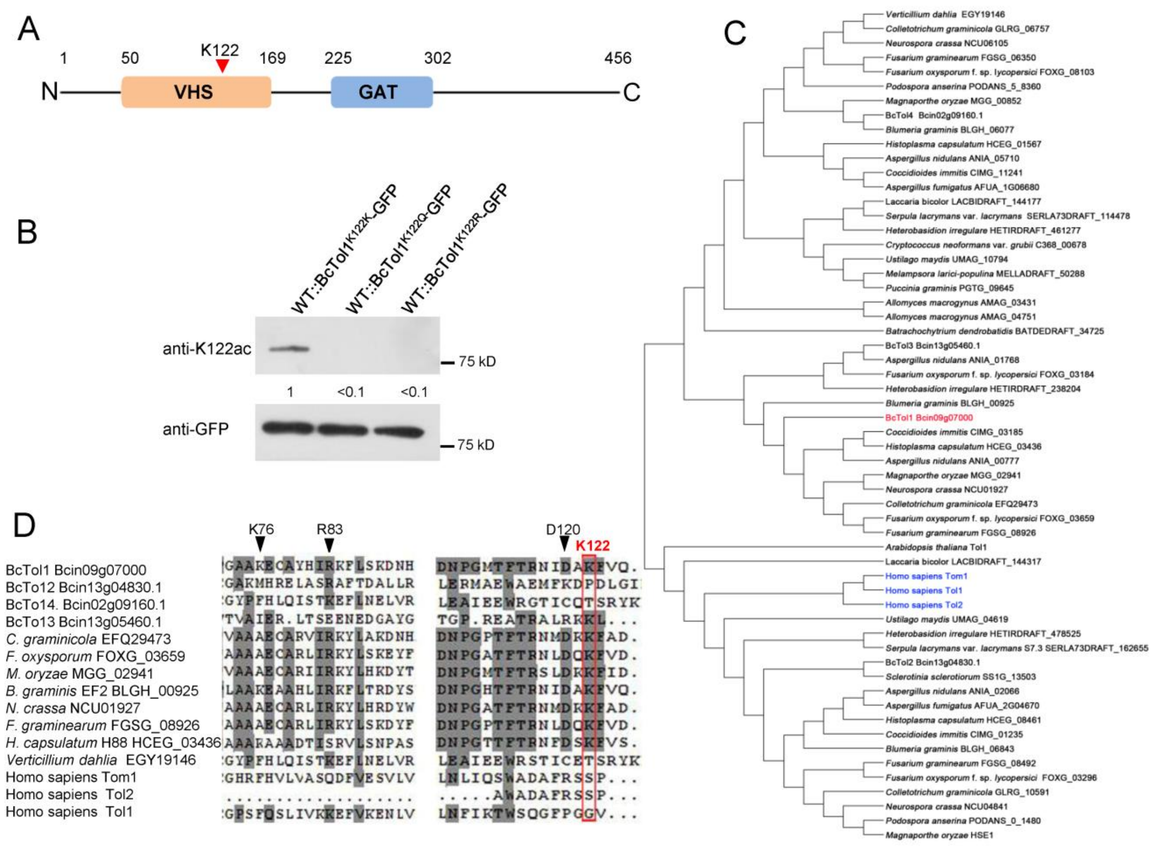

Figure S1. Acetylation of BcTol1 and phylogenetic analysis of its homologs. Related to Figure 1.

(A) The domain organization of BcTol1. The VHS and GAT domains, and the acetylated K122 residue were indicated.

(B) Acetylation of WT and K122 mutant BcTol1 proteins. BcTol1-GFP proteins pulled down from the indicated samples with GFP-Trap beads were probed with anti-K122ac and anti-GFP antibodies, respectively.

(C) Phylogenetic analysis of putative Tom1 and Tol proteins from *Homo sapiens*, *Arabidopsis thaliana* and 21 selected fungi. Maximum likelihood method was used to construct the phylogenetic tree.

(D) Alignment of the amino acid sequences of the VHS domain in putative Tom1 and Tol proteins from different species using DNAMAN6.0. K76, R83, D120 and K122 were indicated.

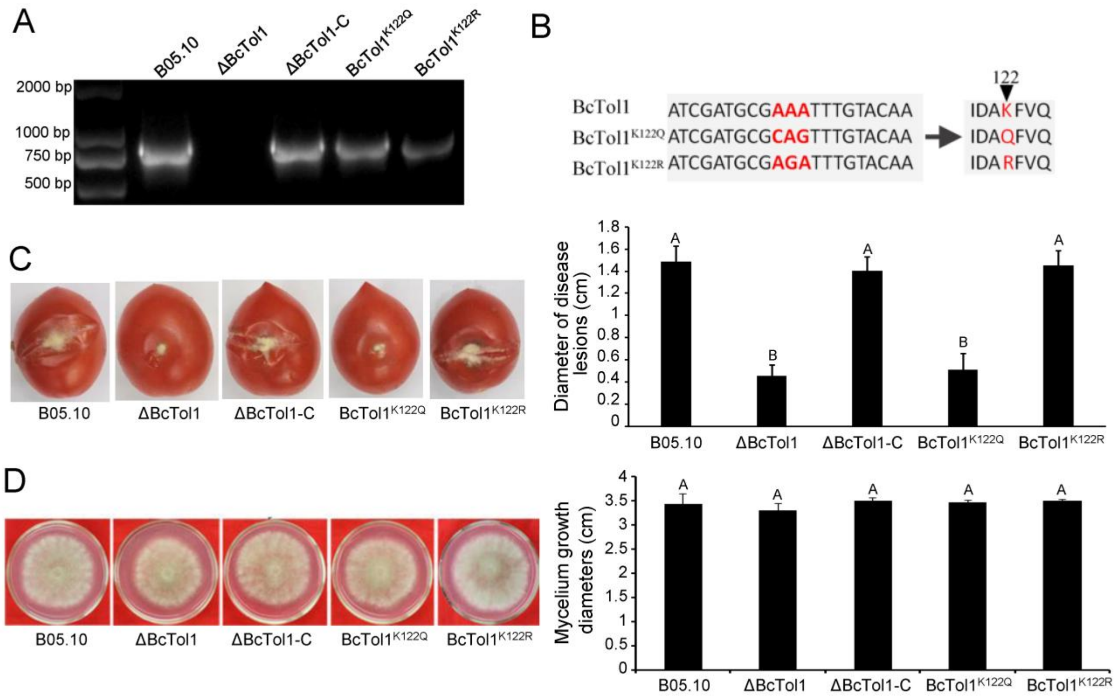

Figure S2. Generation of *BcTol1* mutant strains and phenotypic analysis. Related to Figure 1.

(A) PCR analysis to determine ΔBcTol1, ΔBcTol1-C, BcTol1^{K122Q} and BcTol1^{K122R} mutant strains. Genomic DNA was analyzed by PCR with the primer pairs indicated in Table S2.

(B) Sequence analysis showing mutation of K122 to Q or R.

(C) Virulence of B05.10 and BcTol1 mutant strains on tomato fruits. Photographs were taken 3 days after inoculation and the diameter of disease lesions was measured for 10 infected fruits.

(D) Morphology and mycelial diameter of B05.10 and BcTol1 mutant strains. Photographs were taken 60 h after incubation. The bars denote the standard errors of four replicates.

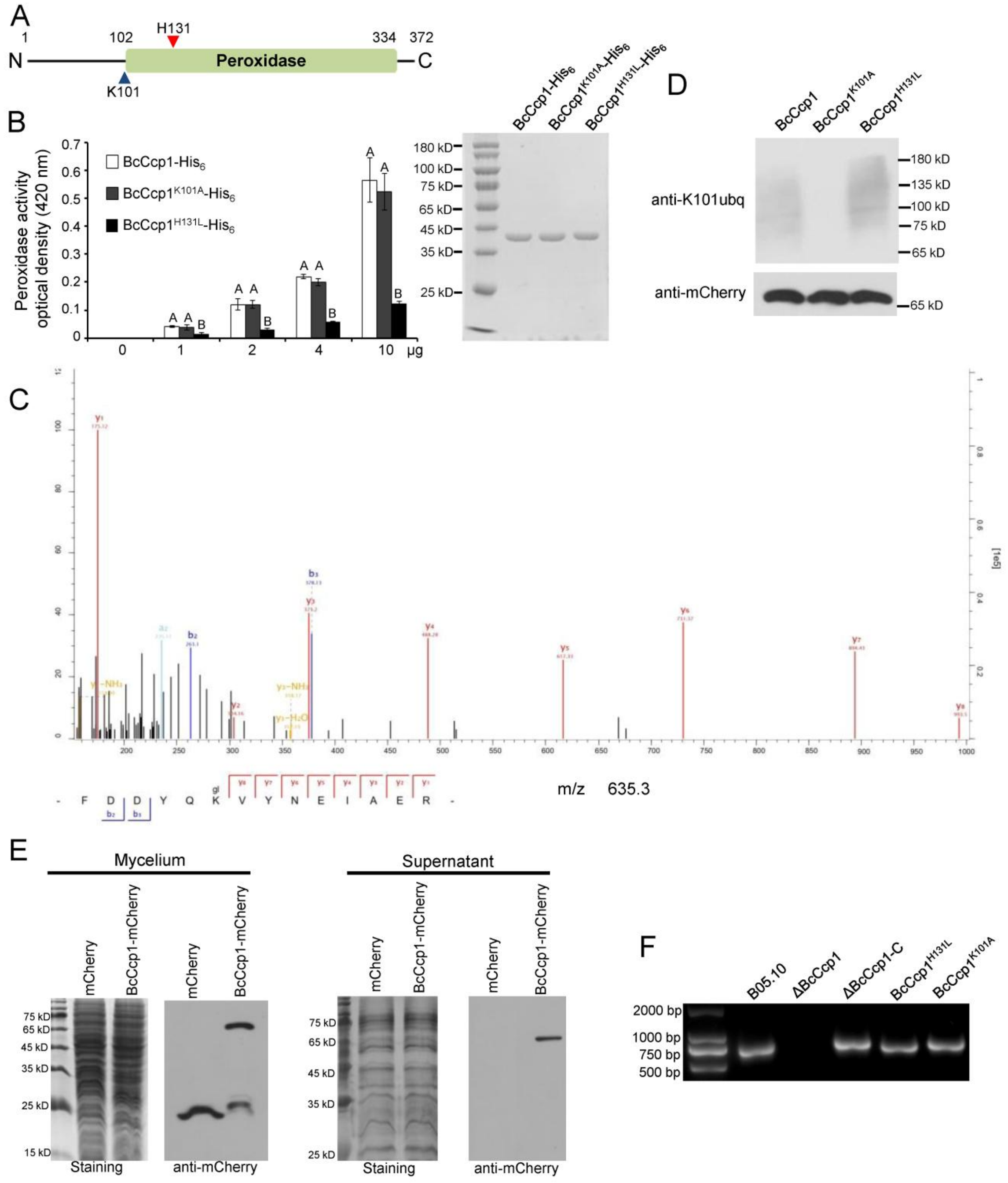

Figure S3. Activity, ubiquitination and secretion of BcCcp1, and generation of *BcCcp1* mutant strains. Related to Figures 2 and 3.

(A) The domain organization of BcCcp1. The peroxidase domain, the ubiquitinated K101 residue, and the core catalytic residue, H131, were indicated.

(B) Activity of WT and mutant BcCcp1 proteins. Peroxidase activity was determined by the ABTS oxidation assay with the purified BcCcp1-His_6 proteins. A coomassie brilliant blue (CBB) stained SDS-PAGE gel shows the purity of the WT and mutant BcCcp1 proteins.

(C) Annotation of representative tandem mass spectra from trypsin-digested BcCcp1-mCherry in *B. cinerea* depicting K101 ubiquitination.

(D) Ubiquitination of WT and mutant BcCcp1 proteins. BcCcp1-mCherry proteins pulled down from the mycelial extract using anti-mCherry antibody agarose beads were probed by anti-K101ubq and anti-mCherry antibodies, respectively.

(E) Secretion of BcCcp1. Conidia of the indicated strains were inoculated into 1/10 YEPD medium in the presence of two-week old tomato seedlings. Twenty-four hours after inoculation, total proteins extracted from the mycelia or culture supernatant were probed with anti-mCherry antibody. CBB or silver staining shows protein loading to each lane.

(F) PCR analysis to determine ΔBcCcp1, ΔBcCcp1-C, BcCcp1^{K122Q} and BcCcp1^{K122R} mutant strains. Genomic DNA was analyzed by PCR with the primer pairs indicated in Table S2.

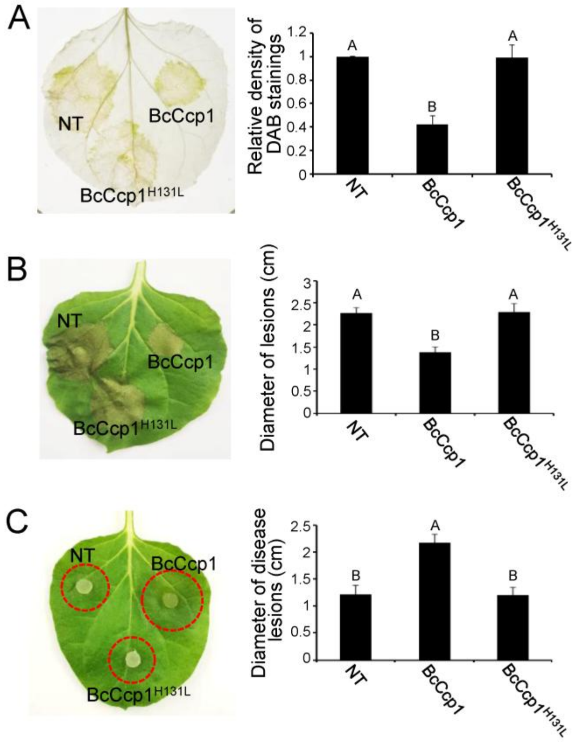

Figure S4. Effect of BcCcp1 on BAX caused H_2O_2 accumulation and cell death, and the infection of *Phytophthora capsici* in tobacco leaves. Related to Figures 3.

(A) Effect of BcCcp1 on H_2O_2 accumulation. Recombinant BcCcp1-His$_6$ or BcCcp1^{H131L}-His$_6$ was infiltrated into *N. benthamiana* leaves together with pTRV2-BAX containing *A. tumefaciens*, and DAB staining was performed at 36 hpi.

(B) Effect of BcCcp1 on cell death. Recombinant BcCcp1-His$_6$ or BcCcp1^{H131L}-His$_6$ was infiltrated into *N. benthamiana* leaves together with pTRV2-BAX containing *A. tumefaciens*. Photographs were taken 3 days after injection.

(C) Effect of BcCcp1 on *Phytophthora capsici* infection. Recombinant BcCcp1-His$_6$ or BcCcp1^{H131L}-His$_6$ was infiltrated into *N. benthamiana* leaves, and *P. capsici* was then inoculated. Photographs were taken 2 days after inoculation and diameter of disease lesions were measured. The presence of different letters above the mean values of three

replicates indicates a significant difference between different samples ($P < 0.05$, ANOVA). NT means no infiltration.

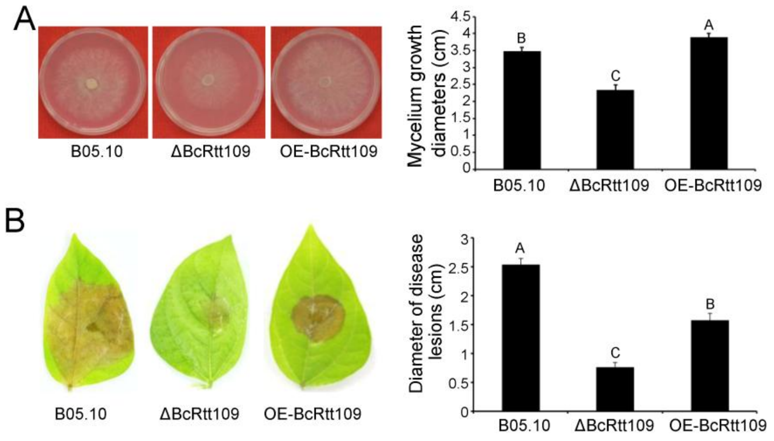

Figure S5. Phenotypic analysis of the ΔBcRtt109 and the OE-BcRtt109 strains. Related to Figures 4.

(A) Morphology and mycelial diameter of the B05.10, the ΔBcRtt109 and the OE-BcRtt109 strains. Photographs were taken 60 h after incubation. The bars denote the standard errors of three replicates.

(B) Virulence of the B05.10, the ΔBcRtt109 and the OE-BcRtt109 strains on mung bean leaves. Photographs were taken 4 days after inoculation and the diameter of disease lesions was measured for 10 infected leaves of each strain.

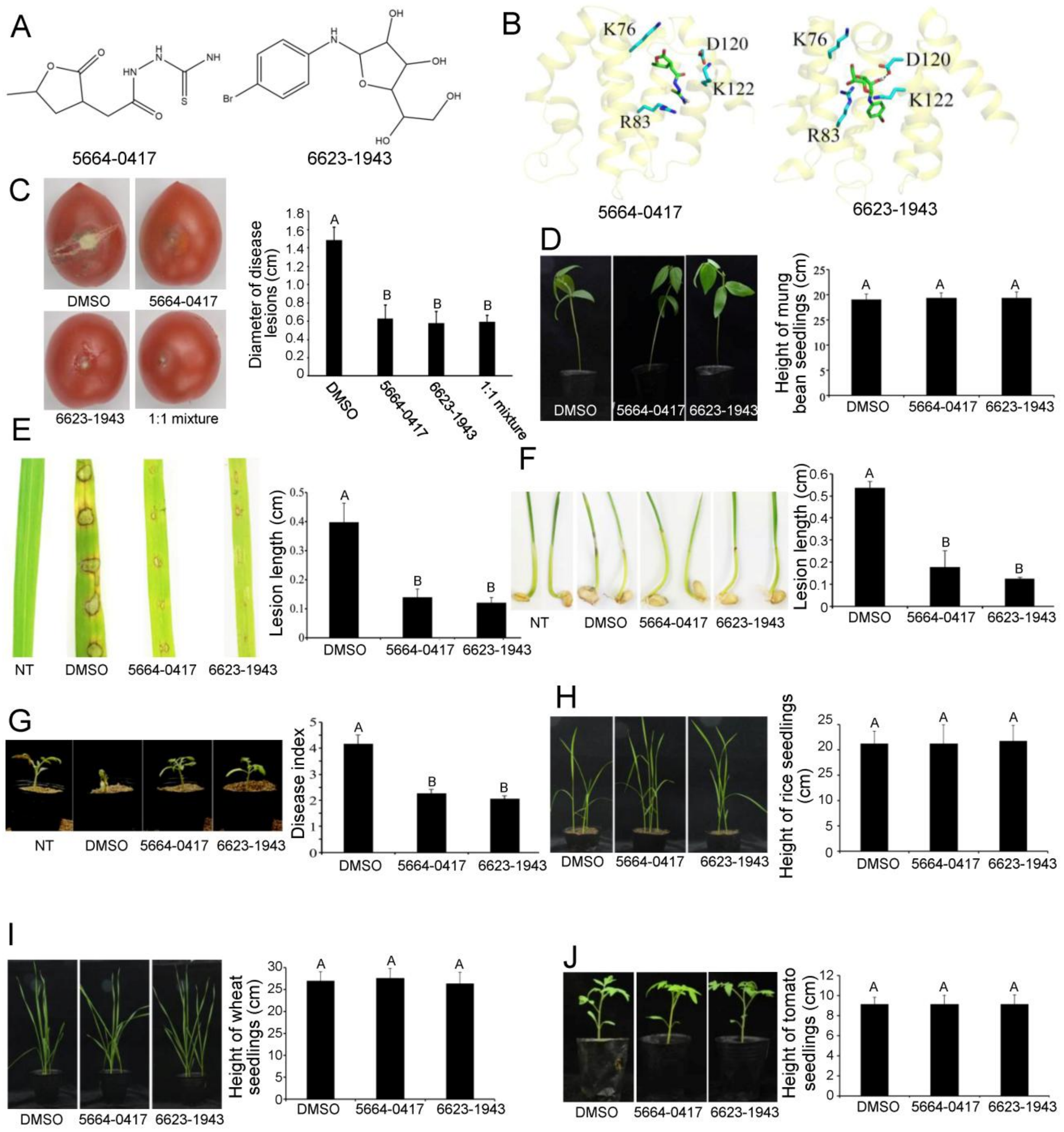

Figure S6. Effect of BcTol1 targeting chemicals on fungal pathogens and safety test. Related to Figures 5.

(A) Structure of 5664-0417 and 6623-1943.

(B) Re-docking showing the binding regions of 5664-0417 and 6623-1943 to BcTol1.

(C) Virulence of *B. cinerea* on tomato fruits with or without the application of 5664-0417, 6623-1943 or 1:1 mixture of the two chemicals.

(D) Safety test of the chemicals on mung bean seedlings.

(E) Virulence of *M. oryzae* on rice leaves with or without the application of 5664-0417 or 6623-1943. *M. oryzae* inoculation was performed simultaneously with treatment with 10 μM 5664-0417 or 6623-1943. Photographs were taken 5 days after inoculation and the length of disease lesions were measured.

(F) Virulence of *F. graminearum* on wheat coleoptiles with or without the application of 5664-0417 or 6623-1943. *F. graminearum* inoculation was performed simultaneously with treatment with 10 μM 5664-0417 or 6623-1943. Photographs were taken three days after inoculation and the diameter of disease lesions were measured.

(G) Virulence of *F. oxysporum* on tomato seedlings with or without the application of 5664-0417 or 6623-1943. *F. oxysporum* inoculation was performed simultaneously with treatment with 10 μM 5664-0417 or 6623-1943. Photographs were taken three weeks after inoculation and disease index was measured.

(H) Safety test of the chemicals on rice seedlings.

(I) Safety test of the chemicals on wheat seedlings.

(J) Safety test of the chemicals on tomato seedlings. For (B), (F)-(H), photographs were taken 2 weeks after spraying of 100 μM of the chemicals or DMSO and seedling heights were measured. The presence of different letters above the mean values of three replicates indicates a significant difference between different samples ($P < 0.05$, ANOVA).

Table S1. Primers used in the study. Related to STAR Methods.

Primer	Sequence(5'-3')	Relevant characteristics
BcTol1-up-F	GTTCAGGCTCTCTTGAAGAA	Amplify *BcTol1* upstream fragment for the construction of *BcTol1* deletion mutants
BcTol1-up-R	**GACCTCCACTAGCTCCAGCCAAGCC** ATATTCTTCATGGCT	
BcTol1-down-F	**ATAGAGTAGATGCCGACCGCGGGTT** ATCGTCAATGCTTCATCCC	Amplify *BcTol1* downstream fragment for the construction of *BcTol1* deletion mutants
BcTol1-down-R	CTGCTGATGCTGTGAGTTCA	
HPH-F	GGCTTGGCTGGAGCTAGTGGAGGTC	Amplify the hygromycin resistant gene *HPH*
HPH-R	AACCCGCGGTCGGCATCTACTCTAT	
BcTol1-K-F	ATACCGTGATCTTGAGGGGAA	Amplify the *BcTol1* deletion castle: up-HPH-down
BcTol1-K-R	ATGGAAGGTAAGGCTGCTAAG	
BcTol1-in-F	TTGAATTTGACTGACCATGGA	Identification of *BcTol1* deletion transformants
BcTol1-in-R	AGGGGGAAGGCGATGAGTA	
BcTol1-out-F	TGATTGCCACATGATGGCTT	Identification of *BcTol1* deletion transformants
BcTol1-out-R	TATCTGACACCGCAACACAA	
BcTol1-C-F	agaagcttAGGATGAGGCGTGTTTGTTT	Amplify full *BcTol1* including its up and dowm fragment for complement of the *BcTol1* deletion mutant
BcTol1-C-R	atgaattcGCATCGTCTTCACTGTTCTTG	
BcTol1-K122R-F	AATATCGATGCGAGATTTGTACAAA	Introduce K122R to BcTol1
BcTol1- K122R-R	TTTGTACAAATCTCGCATCGATATT	
BcTol1- K122Q-F	AATATCGATGCGCAGTTTGTACAAA	Introduce K122Q to BcTol1
BcTol1- K122Q-R	TTTGTACAAACTGCGCATCGATATT	
BcTol1-VHS-F	AGACCCTCAGCACGATACACCCGAGCA AGAGAAAATGATG	Amplify *BcTol1* without VHS domain
BcTol1-VHS-R	CATCATTTTCTCTTGCTCGGGTGTATCG TGCTGAGGGTCT	
BcTol1-GFP-F	**TGCAGCTGTGGAGCCGCATTCCC** AGGATGAGGCGTGTTTGTTT	Amplify full length *BcTol1* including its promoter for pNAN-OGG vector construction
BcTol1-GFP-R	**TACTTACCTCACCCTTGGAAACCAT** GTATCGATAGACAGGTTGC	
olic-F	**CTATAGGGCGAATTGGGTACTCAAAT TGGTT** TGCAGCTGTGGAGCCGCATT	Amplify full length olic promoter from pNAN-OGG for phz126-olicP-Flag vector construction
olic-R	**CTTTATAATCACCGTCATGGTCTTTG TAGTC** CTCGAGTTGGATCGATTGTGATGTGATG G	
BcTol1-Flag-F	**CCATCACATCACAATCGATCCAACC** ATGAAAGCCATGAAGAATATGG	Amplify full length BcTol1 for phz126-olicP-Flag vector construction
BcTol1- Flag -R	**CTTTATAATCACCGTCATGGTCTTTG TAGTC**GTATCGATAGACAGGTTGC	

BcActin-F	CATGGCTGGTCGTGATTTGA	*BcActin* for qPCR
BcActin-R	GAGGATTGACTGGCGGTTTG	
BcTol1-ex-F	CAGCACCGGACAATGATAC	*BcTol1* for qPCR
BcTol1-ex-R	CTGGTGGAGGAATCGGTG	
BcCcp1-ex-F	ATAAGGACGCTGCTGC	*BcCcp1* for qPCR
BcCcp1-ex-R	TTCCAGCTCCACTTCTCA	
BcRtt109-ex-F	ACTTGTTAGAAAGACCTGAG	*BcRtt10*9 for qPCR
BcRtt109-ex-R	TTGGTTCCTGTGACTAATTG	
BcTol1-BD-F	**GAGGACCTGCATATG**ATGAAAGCCA TGAAGAATATGGGCA	Amplify full length *BcTol1* for Y2H vector construction
BcTol1-BD-R	**CTCCATGGCCATATG**GTATCGATAGA CAGGTTGC	
Ubq-AD-F	**GTACCAGATTACGCTCATATG**ATGCA GATCTTCGTC	Amplify full length ubiquitin for Y2H vector construction
Ubq-AD-R	**ACTGGCCTCCATGGCCATATG**TTATT GACCACCACGAAG	
BcCcp1-AD-F	**GTACCAGATTACGCTCATATG**ATGGC ATCCGCTACTCG	Amplify full length *BcCcp1* for Y2H vector construction
BcCcp1-AD-R	**ACTGGCCTCCATGGCCATATG**CTAG GCAGTAGTAGGCTTGAATT	
Ubq- BcCcp1- F	TTCGTGGTGGTCAAATGGCATCCGCTA CTCG	Fusion of ubiquitin and BcCcp1
Ubq-BcCcp1- R	CGAGTAGCGGATGCCATTTGACCACCA CGAA	
BcCcp1-up-F	GCCAATGTGAAACGATGATTG	Amplify *BcCcp1* upstream fragment for the construction of *BcCcp1* deletion mutants
BcCcp1-up-R	**GACCTCCACTAGCTCCAGCCAAGCC** AGTAGCGGATGCCATTTTGA	
BcCcp1-down-F	**ATAGAGTAGATGCCGACCGCGGGTT** CTTTGGGGTGCAAAATTGGT	Amplify *BcCcp1* downstream fragment for the construction of *BcCcp1* deletion mutants
BcCcp1-down-R	ATGGTCCCTTCCCCTCAGAT	
BcCcp1-K-F	TCAAGTGGGTCGAGATTTGCT	Amplify the *BcCcp1* deletion castle: up-HPH-down
BcCcp1-K-R	ACATCACCCCTTACTTCCTCA	
BcCcp1-in-F	AATCAACACCTTTCAGATCC	Identification of *BcCcp1* deletion transformants
BcCcp1-in-R	CGTCCTTATCTTGTCTACCT	
BcCcp1-out-F	CAACCCATTTATCAAAATGGC	Identification of *BcCcp1* deletion transformants
BcCcp1-out-R	GACTTGGAGGTCTTGTCTTCG	
BcCcp1-C-F	**ATCGATAAGCTTGATATCGAATTC**GC CAATGTGAAACGATGATT	Amplify full *BcCcp1* including its up and down fragment for complement of the *BcCcp1* deletion mutant
BcCcp1-C-R	**TAGAACTAGTGGATCCCCCGGG**ATG GTCCCTTCCCCTCAGAT	
BcCcp1-mcherry-Na-F	**TGCAGCTGTGGAGCCGCATTCCC**GC CAATGTGAAACGATGATT	Amplify full length *BcCcp1* including its promoter for pNAB-OCT vector construction
BcCcp1-mcherry-R	**TACTTACCTCGCCCTTGCTTACCAT** GGCAGTAGTAGGCTTGAATTTCA	
BcCcp1-Flag-F	**CCATCACATCACAATCGATCCAACC** ATGGCATCCGCTACTCG	Amplify full length *BcCcp1* for phz126-olicP-Flag vector

BcCcp1- Flag -R	**CTTTATAATCACCGTCATGGTCTTTG TAGTC**GGCAGTAGTAGGCTTGAATTTCA	construction
BcCcp1-28a-F	**AGCAAATGGGTCGCGGATCCGAATTC** ATGGCATCCGCTACTCG	Amplify full length *BcCcp1* for Pet-28a vector construction
BcCcp1-28a-R	**TCAGTGGTGGTGGTGGTGGTGCTCG AGCTAGG** GGCAGTAGTAGGCTTGAATTTCA	
BcCcp1-K101A-F	GATTACCAAGCCGTTTAC	Introduce K101A to BcCcp1
BcCcp1-K101A-R	GTAAACGGCTTGGTAATC	
BcCcp1-H131L-F	TGGCGTGGTTGTGCAGC	Introduce H131L to BcCcp1
BcCcp1-H131L-R	GCTGCACAACCACGCCA	
BcRtt109-Flag-F	**CCATCACATCACAATCGATCCAACC**ATGGCTACTCGAGGGAGTTTTGG	Amplify full length *BcRtt109* for phz126-olicP-Flag vector construction
BcRtt109-Flag-R	**CTTTATAATCACCGTCATGGTCTTTG TAGTC**CAGGGCTGGCGCATTTGG	
BcRtt109-GFP-F	**TGCAGCTGTGGAGCCGCATTCCC**TAGCGTACAATGAAGTAGCT	Amplify full length *BcRtt109* including its promoter for pNAN-OGG vector construction
BcRtt109- GFP -R	**TACTTACCTCACCCTTGGAAACCAT**CAGGGCTGGCGCATTTGG	
BcRtt109-GST-F	TGGTTCCGCGTGGATCCATGGCTACTC GAGGGAGTTTTGG	Amplify full length *BcRtt109* for pGEX-4T-2 vector construction
BcRtt109-GST-R	GGAATTCCTGGGGATCCTTAGTTCTTTA CCTTTTTTCTAATCATACCAGC	
BcTol1-28a -F	**AGCAAATGGGTCGCGGATCCGAATTC**ATGAAAGCCATGAAGAATATGG	Amplify full length of *BcTol1* for Pet-28a vector construction
BcTol1-28a -R	**TCAGTGGTGGTGGTGGTGGTGCTCG AG**GTATCGATAGACAGGTTGCTTAC	
BcTol1-28aV-F	**GAAGGAGATATACCATGGCA**ATGAAAGCCATGAAGAATATGG	Amplify VHS domain of *BcTol1* for pET-28a vector construction
BcTol1-28aV-R	**GTGGTGGTGG TG CTCGAG**GTACGCTTGTACACTTCTGCT	
K76A-R83A-F	GGAGCTGCGGCCGAATGCGCCTACCAT ATTGCCAAGTTCCTC	Introduce K76A and R83A to BcTol1
K76A-R83A-R	GAGGAACTTGGCAATATGGTAGGCGCA TTCGGCCGCAGCTCC	
K122Q-D120A-F	AGGAATATCGCCGCGCAGTTTGTACAA	Introduce K122Q and D120A to BcTol1
K122Q-D120A-R	TTGTACAAACTGCGCGGCGATATTCCT	
