# Supplementary material for: Broad-spectrum chemicals block ROS detoxification to prevent plant fungal invasion
Source: Curr Biol. Author manuscript; Available in PMC 2022 Oct 13. (PMC7613639; doi:10.1016/j.cub.2022.07.022)
Supplement: Supplementary Information [file EMS152247-supplement-Supplementary_Information.zip › 1-s2.0-S096098222201123X-mmc3.pdf]

Current Biology

Article

Broad-spectrum chemicals block ROS detoxification to prevent plant fungal invasion

Graphical abstract

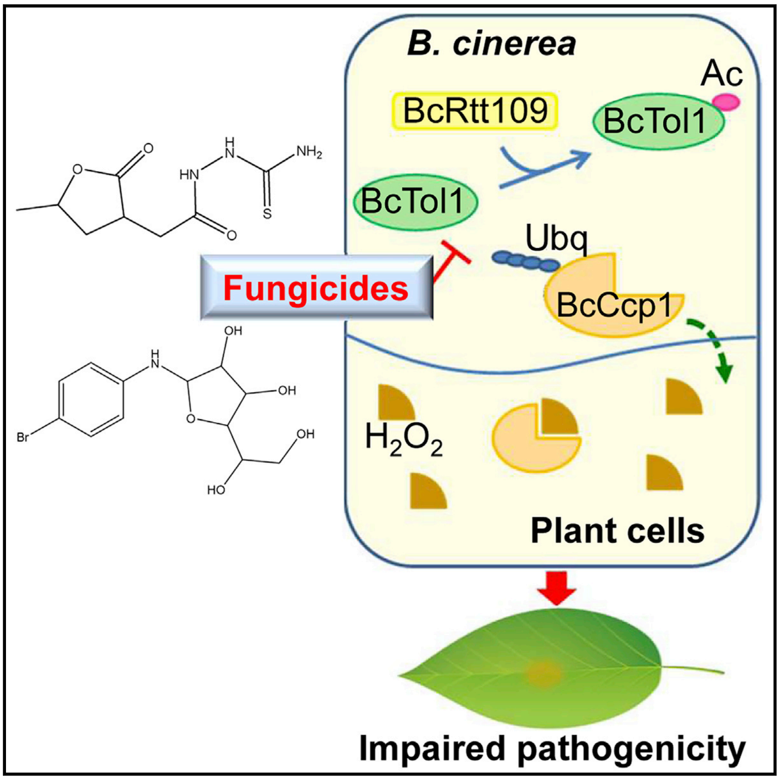

Authors

Qianqian Yang, Jinguang Yang, Yameng Wang, Juan Du, Jianan Zhang, Ben F. Luisi, Wenxing Liang

Correspondence

wliang1@qau.edu.cn

In brief

Plants produce reactive oxygen species (ROS) to restrict pathogen invasion. Yang et al. reveal a mechanism of ROS detoxification employed by *Botrytis cinerea* to facilitate fungal invasion. They identify broad-spectrum compounds that block ROS detoxification and provide a class of potential fungicides to control a wide range of plant diseases.

Highlights

- The BcCcp1 peroxidase secreted by *Botrytis cinerea* eliminates plant-produced H_2O_2
- The ubiquitin receptor BcTol1 modulates BcCcp1 secretion to promote fungal invasion
- Two BcTol1-targeting compounds impede BcCcp1 secretion and *B. cinerea* infection
- The chemicals serve as potential drugs to control diverse plant diseases

Yang et al., 2022, Current Biology *32*, 1–12
September 26, 2022 © 2022 The Author(s). Published by Elsevier Inc.
https://doi.org/10.1016/j.cub.2022.07.022

CellPress

Please cite this article in press as: Yang et al., Broad-spectrum chemicals block ROS detoxification to prevent plant fungal invasion, Current Biology (2022), https://doi.org/10.1016/j.cub.2022.07.022

Current Biology

CellPress
OPEN ACCESS

Article

Broad-spectrum chemicals block ROS detoxification to prevent plant fungal invasion

Qianqian Yang,[1,5] Jinguang Yang,[2,5] Yameng Wang,[1] Juan Du,[3] Jianan Zhang,[1] Ben F. Luisi,[4] and Wenxing Liang[1,6,*]
[1]College of Plant Health and Medicine, Engineering Research Center for Precision Pest Management for Fruits and Vegetables of Qingdao, Shandong Engineering Research Center for Environment-Friendly Agricultural Pest Management, Shandong Province Key Laboratory of Applied Mycology, Qingdao Agricultural University, Qingdao 266109, China
[2]Tobacco Research Institute of CAAS, Qingdao 266100, China
[3]College of Life Sciences, Qingdao Agricultural University, Qingdao 266109, China
[4]Department of Biochemistry, University of Cambridge, Cambridge CB2 1GA, UK
[5]These authors contributed equally
[6]Lead contact
*Correspondence: wliang1@qau.edu.cn
https://doi.org/10.1016/j.cub.2022.07.022

SUMMARY

Plant diseases cause a huge impact on food security and are of global concern. While application of agrochemicals is a common approach in the control of plant diseases currently, growing drug resistance and the impact of off-target effects of these compounds pose major challenges. The identification of pathogenicity-related virulence mechanisms and development of new chemicals that target these processes are urgently needed. One such virulence mechanism is the detoxification of reactive oxygen species (ROS) generated by host plants upon attack by pathogens. The machinery of ROS detoxification might therefore serve as a drug target for preventing plant diseases, but few anti-ROS-scavenging drugs have been developed. Here, we show that in the model system *Botrytis cinerea* secretion of the cytochrome *c*-peroxidase, BcCcp1 removes plant-produced H_2O_2 and promotes pathogen invasion. The peroxidase secretion is modulated by a Tom1-like protein, BcTol1, through physical interaction. We show that BcTol1 is regulated at different levels to enhance the secretion of BcCcp1 during the early infection stage. Inactivation of either BcTol1 or BcCcp1 leads to dramatically reduced virulence of *B. cinerea*. We identify two BcTol1-targeting small molecules that not only prevent *B. cinerea* invasion but also have effective activity against a wide range of plant fungal pathogens without detectable effect on the hosts. These findings reveal a conserved mechanism of ROS detoxification in fungi and provide a class of potential fungicides to control diverse plant diseases. The approach described here has wide implications for further drug discovery in related fields.

INTRODUCTION

Plant diseases due to the infection of plant pathogens cause global threats to agricultural sustainability and food security.[1,2] Among all the disease management strategies implemented, application of pesticides is the most common management practice. Due to the limited number of efficient fungicides available and their frequent use, drug resistance has widely occurred.[3,4] Broad-spectrum agrochemicals with anti-fungal properties have undesirable off-target effects, while site-specific inhibitors carry the risk of high resistance developing.[5,6] This trade-off has urged plant pathologists to obtain new and better fungicide targets by identifying the pathways involved in host colonization and immune evasion. Septin GTPases and pyruvate kinase have been identified as novel targets, and the corresponding inhibitors have been discovered.[7,8] However, so far, only a limited number of potential drug targets have been identified in plant fungal pathogens. Even for these targets, few drugs have been developed and approved for use. Therefore, identification of new targets for disease intervention and development of previously unused chemicals are urgently needed.

Plants have evolved elaborate and sensitive protection systems to combat pathogens. Oxidative burst, a rapid plant defense reaction after pathogen attack, is a critical and effective component of plant immunity.[9,10] Reactive oxygen species (ROS), including superoxide and the precursor H_2O_2, not only result in hypersensitive response (HR) and local cell death to block pathogen colonization but also act as signaling molecules to activate the expression of defense-related genes.[11] In order to infect successfully, pathogens need to overcome oxidative burst by either incapacitating ROS production[12] or detoxifying ROS generated by the host.[13] One key way of ROS detoxification is by enzymatic mechanisms. Peroxidases (EC1.11.1.x), a group of evolutionarily conserved enzymes that mediate electron transfer from H_2O_2 and organic peroxide to various electron acceptors, are workhorses for the fungal antioxidant defense system, and play crucial roles in virulence of plant fungal pathogens.[14–17] Several peroxidases capable of breaking down H_2O_2 have been identified in the fungal pathogen *Botrytis cinerea*, and BcCcp1, a cytochrome

This is an open access article under the CC BY-NC-ND license (http://creativecommons.org/licenses/by-nc-nd/4.0/).

c-peroxidase (Ccp) protein is involved in pathways contributing to host specific pathogenicity of this pathogen.[18,19] However, to date, few anti-ROS detoxification drugs have been developed to control plant fungal diseases.

To maintain the function of cellular organizations, proteins must be correctly delivered to their target subcellular compartments. Target of Myb 1 (Tom1) and Tom1-like (Tol) proteins, widely distributed in eukaryotes but absent in *Saccharomyces cerevisiae*, are evolutionarily ancient ubiquitin receptors that function in compartment delivery. They contain two conserved domains, an N-terminal VHS (Vps27/Hrs/STAM) domain and a central GAT domain,[20,21] which are both required for the binding of ubiquitin. These proteins, acting together with or replacing the endosomal sorting complex required for transport machinery, are capable of recognizing and sorting of ubiquitinated cargoes. In higher plants and mammalians, Tom1 and Tol proteins are involved in a variety of physiological processes, which crosstalk with their endosomal cargo sorting function.[22–25] However, the Tom1 family has not yet been characterized in filamentous fungi, and its regulatory role in protein secretion and virulence of plant fungal pathogens remain elusive.

B. cinerea is a necrotroph that causes gray mold during both pre- and post-harvest, leading to huge crop losses.[26–28] Our previous proteomics studies identified BcTol1 (Bcin09g07000), a Tom1-like protein in *B. cinerea*, as a putative modified protein with acetylation on the lysine residue, 122, which is located in the N-terminal VHS domain.[29] In this study, we find that BcTol1 physically associates with the ubiquitinated BcCcp1, via its VHS domain. The secretion of BcCcp1 is enhanced during the early infection stage through upregulation of BcTol1 at the transcriptional and translational level and decreased acetylation on K122. By eliminating plant-produced H_2O_2, BcCcp1 contributes to successful host invasion of *B. cinerea*. Removal of BcTol1 or BcCcp1, or mutation of the ubiquitinated residue, K101, in BcCcp1 dramatically impairs *B. cinerea* pathogenicity. Based on the predicted structure of the VHS domain, we developed two small chemicals that target BcTol1. Application of these molecules attenuates ROS detoxification, leading to effective inhibition of *B. cinerea* invasion. These chemicals are also effective against several other important plant fungal pathogens with no harm on the hosts, suggesting that they could work as broad-spectrum drugs for crop protection.

RESULTS

BcTol1 is an acetylated protein

The putative lysine residue, 122, is located in the VHS domain of BcTol1 (Figure S1A). To confirm acetylation of this site, we mutated lysine 122 to glutamine (Q) and arginine (R) to mimic acetylated and unacetylated lysine, respectively,[30,31] and developed a specific antibody against acetylated K122 of BcTol1 (anti-K122ac). BcTol1-GFP, expressed under its native promoter in the B05.10 strain, was pulled down with anti-GFP agarose beads and its acetylation was determined using anti-K122ac. Our results showed that mutation of K122 completely abolished acetylation of BcTol1 (Figure S1B), indicating that K122 is indeed acetylated in this protein.

Systematic analysis identified 57 putative Tom1-like proteins containing both the VHS and GAT domains from 24 species that included 22 fungi, 1 metazoa, and 1 viridiplantae (Figure S1C). Similar to *Homo sapiens* and Arabidopsis, there are several Tol proteins in a variety of fungi. Further alignment showed that lysine at position corresponding to K122 was highly conserved in 9 Tol1 homologous proteins from multiple pathogenic fungi (Figure S1D), suggesting that modification of this residue might play an important regulatory role in the virulence of these plant pathogens.

BcTol1 deacetylation is a requirement for full virulence of *B. cinerea*

To investigate whether BcTol1 is involved in regulating virulence of *B. cinerea*, we assessed the expression level of *BcTol1* during the infection stage by quantitative real-time PCR and western blotting. Both *BcTol1* transcript and its protein product increased dramatically during the early infection stage, reaching the maximum at 24 h post-infection (hpi), and then decreased thereafter (Figures 1A and 1B). In addition, BcTol1 maintained high acetylation at 6 and 12 hpi and then decreased at 24 hpi (Figure 1C). These differential expression patterns suggest that BcTol1 and its acetylation status might play a role in pathogenicity of *B. cinerea*.

To determine the virulence contribution of BcTol1, we generated *BcTol1* knockout mutants (ΔBcTol1) and genetic complementation strains (ΔBcTol1-C) (Figure S2A) and mutated K122 to Q or R under its native promoter in the ΔBcTol1 strain (Figure S2B). Pathogenicity tests on mung bean leaves showed that either inactivation of BcTol1 or replacement of K122 with glutamine impaired virulence of *B. cinerea*, while the ΔBcTol1-C and the arginine mutants behaved very similarly to the wild-type (WT) strains (Figure 1D). Similar virulence defects of the ΔBcTol1 and the $\Delta BcTol1^{K122Q}$ mutant strains were also observed on tomato fruits (Figure S2C). The reduced virulence was not due to decreased growth rate, as ΔBcTol1 only grew slightly slower than B05.10, and acetylation of BcTol1 had no obvious effect on its growth (Figure S2D). These results indicate that BcTol1 and its deacetylation are indispensable for full virulence of *B. cinerea*.

To explore the possible contribution of BcTol1 to ROS detoxification, B05.10 and the corresponding mutants were grown on potato dextrose agar (PDA) plates containing 20 mM H_2O_2. Compared with other strains, both the ΔBcTol1 and the $BcTol1^{K122Q}$ mutants exhibited hypersensitivity to H_2O_2 (Figure 1E). The role of BcTol1 in ROS detoxification was further confirmed by the diaminbezidin (DAB) staining assay. The accumulation of H_2O_2 in mung bean leaves inoculated with the ΔBcTol1 and $BcTol1^{K122Q}$ mutants was ~4-fold higher than those infected with the strains B05.10, ΔBcTol1-C, and $BcTol1^{K122R}$ (Figure 1F). Based on these results, we propose that BcTol1-dependent pathogenicity of *B. cinerea* is tightly associated with ROS detoxification.

BcTol1 associates with and modulates the secretion of a peroxidase, BcCcp1

To assess the ubiquitin binding property of BcTol1, a Y2H assay was performed. As expected, direct interaction between BcTol1 and ubiquitin was observed (Figure 2A), indicating a role of BcTol1 in recognition and further sorting of ubiquitinated cargoes. Mutation of K122 to Q or deletion of the VHS domain

Please cite this article in press as: Yang et al., Broad-spectrum chemicals block ROS detoxification to prevent plant fungal invasion, Current Biology (2022), https://doi.org/10.1016/j.cub.2022.07.022

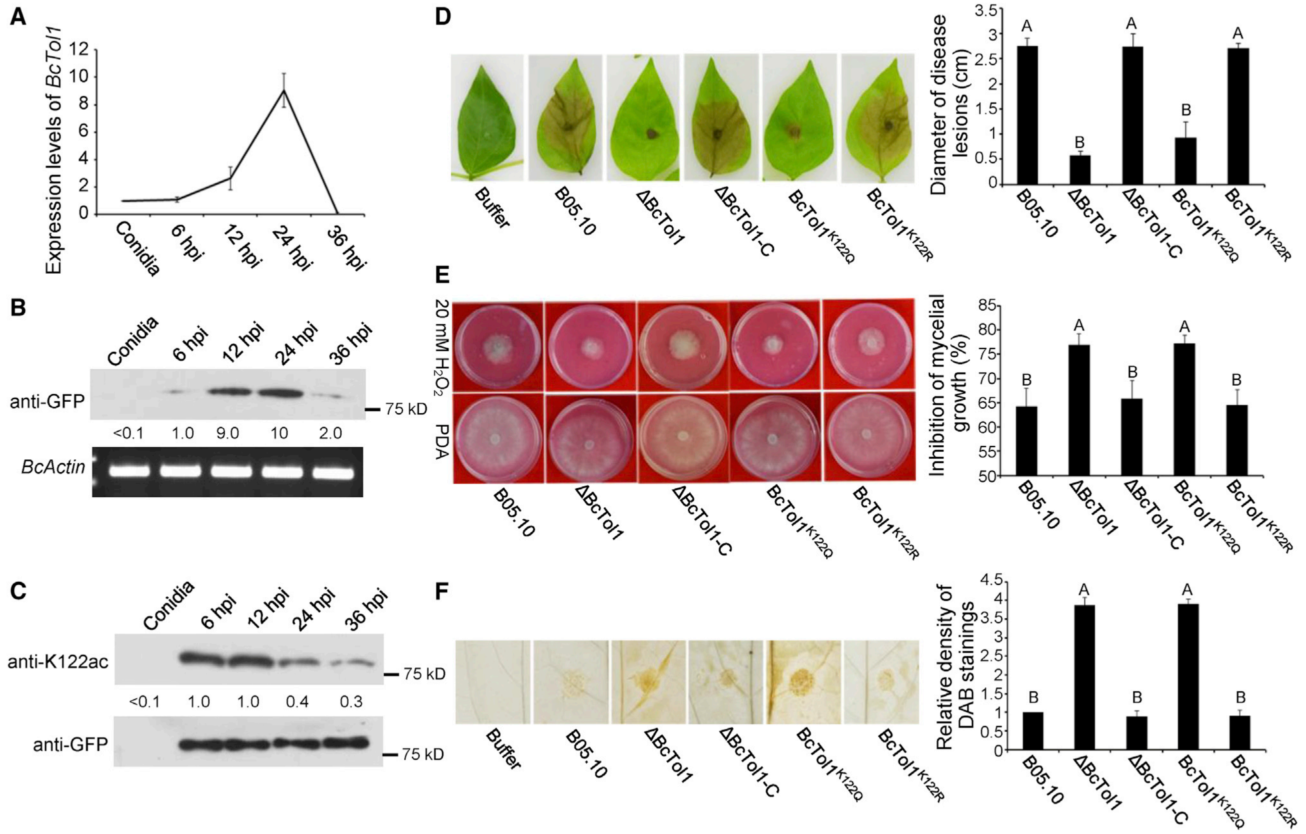

Figure 1. Contribution of BcTol1 to virulence and ROS detoxification of *B. cinerea*

(A) Relative level of *BcTol1* transcript during the infection stage. The expression levels were normalized to that of the *B. cinerea Actin* gene.

(B) Amount of BcTol1 during the infection stage. Total protein was extracted from mung bean leaves inoculated with the BcTol1-GFP strain driven by the native promoter at the indicated times and probed with anti-GFP antibody. *B. cinerea Actin* gene was used as the loading control. The amount of BcTol1 at 6 hpi was set as 1.

(C) Acetylation of BcTol1 during the infection stage. BcTol1-GFP pulled down from the indicated samples was probed with anti-K122ac and anti-GFP antibodies. The amount of acetylated BcTol1 at 6 hpi was set as 1.

(D) Virulence of B05.10 and BcTol1 mutant strains on mung bean leaves. Photographs were taken 4 days after inoculation, and the diameter of disease lesions was measured for 30 infected leaves from 3 replicates of each strain.

(E) Sensitivity of B05.10 and BcTol1 mutant strains to H_2O_2. Photographs were taken 36 h after incubation on PDA medium with or without 20 mM H_2O_2, and the rate of inhibition of mycelial growth was measured for 3 plates of each strain.

(F) DAB staining shows ROS accumulation in mung bean leaves after infection by B05.10 and BcTol1 mutant strains. DAB staining was performed 24 h after inoculation, and the relative density was measured for 10 infected leaves of each strain.

See also Figures S1 and S2.

dramatically decreased the association of BcTol1 with ubiquitin, whereas the R mutant binds as tightly to ubiquitin as the WT form. To identify its ubiquitinated cargoes, we performed coimmunoprecipitation (coIP) using the BcTol1-GFP transformant followed by LC-MS/MS and identified 357 candidate binding partners (Data S1). Since BcTol1 contributes to H_2O_2 tolerance, one putative cytochrome *c*-peroxidase named BcCcp1 (Figure S3A) was selected for further analysis. Using a previously described method,[16,32] we found that His-tagged BcCcp1 purified from *Escherichia coli* indeed had peroxidase activity, while mutation of H131, the putative catalytic site to leucine, led to significantly reduced activity of this enzyme (Figure S3B).

Y2H assays showed that there was no physical interaction between BcCcp1 and BcTol1, while fusion with ubiquitin (Ubq-BcCcp1) facilitated binding of BcCcp1 to BcTol1. To determine the ubiquitinated sites of BcCcp1 in the cells, we purified the BcCcp1-GFP fusion protein from B05.10 and identified lysine 101 as a site of modification by mass spectrometry (Figure S3C). To further prove K101 is ubiquitinated, we developed a specific antibody against ubiquitinated K101 of BcCcp1 (anti-K101ubq). As shown in Figure S3D, mutation of K101, but not H131, completely abolished ubiquitination of BcCcp1, confirming K101 ubiquitination of BcCcp1 *in vivo*. BcTol1-GFP and BcCcp1-FLAG fusion constructs were co-introduced into B05.10 protoplasts, and positive transformants were selected. BcCcp1 was detected in the proteins that eluted from anti-GFP beads using the anti-FLAG antibody, suggesting that BcTol1 interacts with BcCcp1. Either change of K122 to glutamine or removal of the VHS domain impaired the interaction of BcTol1 with BcCcp1, while $BcTol1^{K122R}$ behaved very similarly

Please cite this article in press as: Yang et al., Broad-spectrum chemicals block ROS detoxification to prevent plant fungal invasion, Current Biology (2022), https://doi.org/10.1016/j.cub.2022.07.022

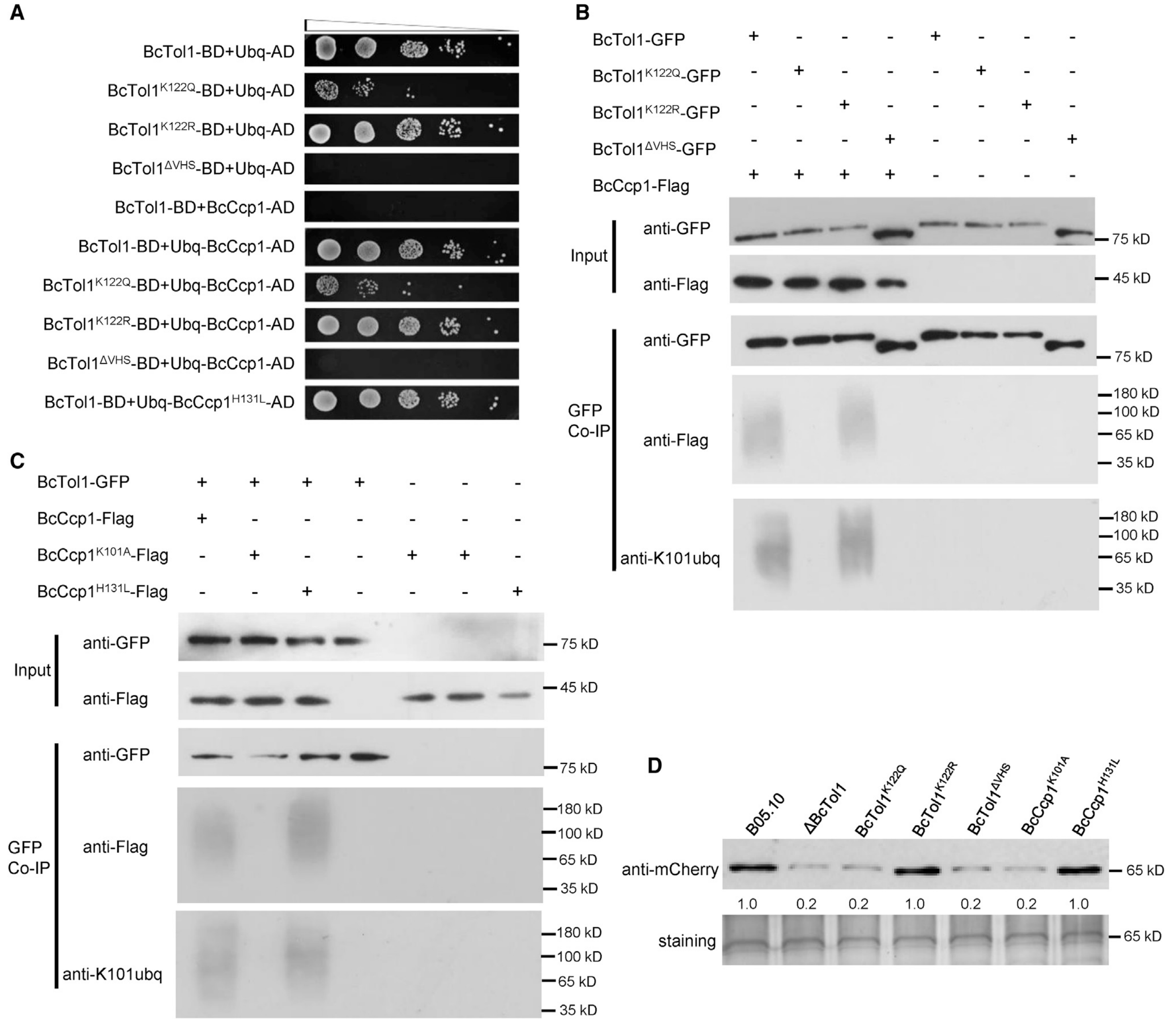

Figure 2. Association of BcTol1 with BcCcp1 and secretion of BcCcp1

(A) Binding of ubiquitin to WT and mutant BcTol1 proteins in yeast cells. The coding sequences of the corresponding proteins were fused with the GAL4-AD or BD domain as indicated. Serial dilutions from cell suspensions of a single yeast colony were shown to represent the strength of interaction. Images were taken 3 days after incubation.

(B) Association of WT and mutant BcTol1 proteins with BcCcp1 *in vivo*.

(C) Association of BcTol1 with WT and mutant BcCcp1 proteins *in vivo*.

For (B) and (C), coIP assays were performed as described in the STAR Methods. Proteins pulled down with GFP-Trap beads were probed with anti-GFP, anti-FLAG, and anti-K101ubq antibodies (bottom). Input proteins were shown by western blotting with anti-GFP and anti-FLAG antibodies (top).

(D) Secretion of BcCcp1 in B05.10 and BcTol1/BcCcp1 mutant strains. Conidia of the indicated strains were inoculated into 1/10 YEPD medium in the presence of 2-week-old tomato seedlings. 24 h after inoculation, total proteins were extracted from culture supernatant and probed with anti-mCherry antibody. The amount of secreted BcCcp1-mCherry in B05.10 was set as 1. Silver staining shows protein loading to each lane.

See also Figure S3 and Data S1.

to the WT protein (Figures 2A and 2B). Not unexpectedly, mutation of K101 to alanine (A) had little effect on the activity of BcCcp1 (Figure S3B) but completely disrupted its association with BcTol1 (Figure 2C). Note that BcCcp1 was detected as a ladder-like smear in the coIP assays, and immunoblot with anti-K101ubq confirmed polyubiquitination of this protein (Figures 2B and 2C).

Some peroxidases could be secreted by plant pathogens.[33] To test secretion of this protein, a recombinant BcCcp1-mCherry fusion protein was expressed under its native promoter in the B05.10 strain, with mCherry serving as a control. Using a method of determining protein secretion in *B. cinerea*,[34] we found that only BcCcp1-mCherry, but not mCherry, was detected in the culture supernatant induced by tomato leaves

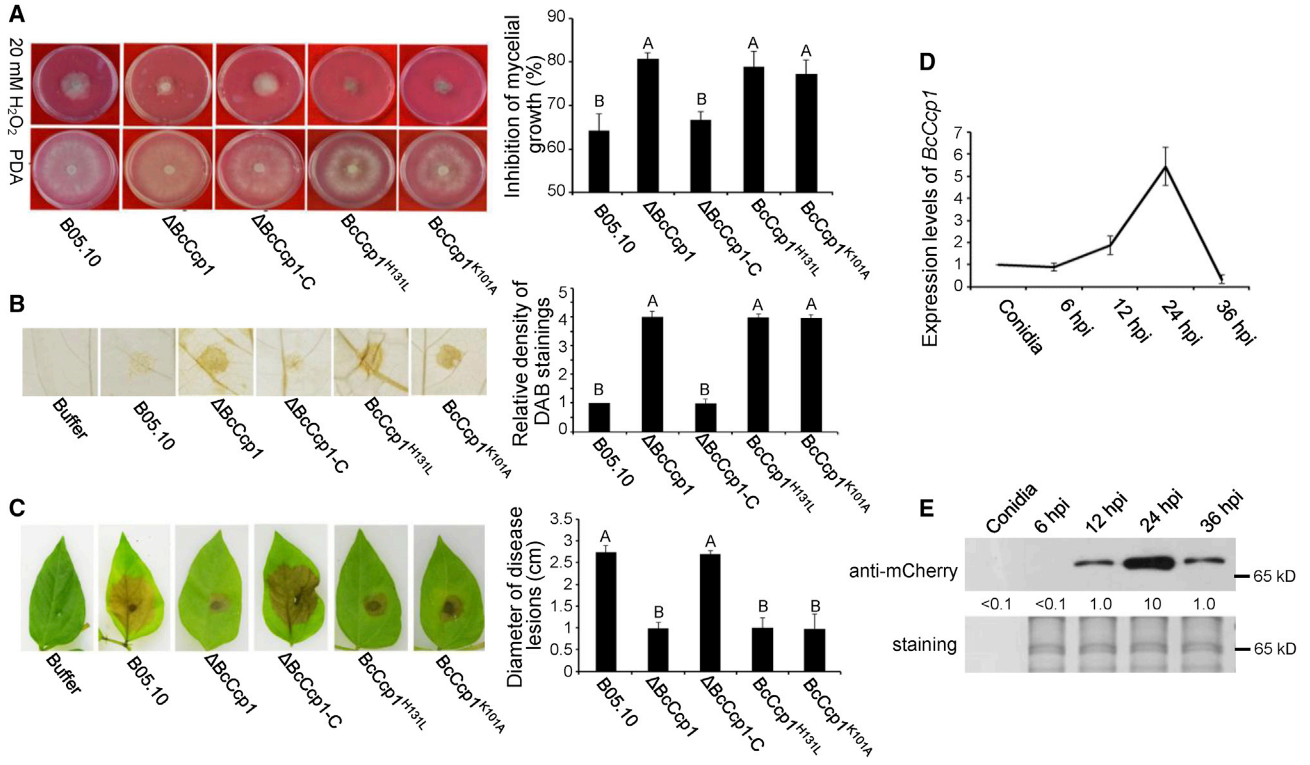

Figure 3. Contribution of BcCcp1 to ROS detoxification and virulence of *B. cinerea*

(A) Sensitivity of B05.10 and BcCcp1-mutant strains to H_2O_2.

(B) DAB staining shows ROS accumulation in mung bean leaves after infection by B05.10 and BcCcp1 mutant strains.

(C) Virulence of B05.10 and BcCcp1 mutant strains on mung bean leaves.

(D) Relative level of *BcCcp1* transcript during the infection stage.

For (A)–(D), the experiments were carried out as in Figure 1.

(E) Amount of secreted BcCcp1 during the infection stage. Total proteins extracted from the culture supernatant after inoculation for the indicated times were probed with anti-mCherry antibody. The amount of BcCcp1 at 12 hpi was set as 1. Silver staining shows protein loading to each lane.

See also Figures S3 and S4.

(Figure S3E), indicating that BcCcp1 is able to be secreted by *B. cinerea*. Due to impaired association, compared with the WT and the K122R mutant strains, BcCcp1 secreted from the ΔBcTol1, ΔVHS, and K122Q mutants was reduced by ~80%. Substitution alanine for K101, which disrupts the association of BcCcp1 with BcTol1, dramatically decreased the amount of BcCcp1 in the supernatant (Figure 2D). In contrast, the H131L-mutant protein, which is enzymatically inactive (Figure S3B), retains the binding activity of BcTol1 (Figure 2C), such that its secretion equals that of the WT protein (Figure 2D). Collectively, these data indicate, first, that BcTol1 modulates the secretion of BcCcp1 through direct interaction, and second, that this association is negatively regulated by K122 acetylation of BcCcp1.

BcCcp1 contributes to ROS detoxification and virulence of *B. cinerea*

To explore its role in ROS detoxification, we generated the *BcCcp1* deletion mutants (ΔBcCcp1) and genetic complementation strains (ΔBcCcp1-C) and mutated K101 to A ($BcCcp1^{K101A}$) and H131 to L ($BcCcp1^{H131L}$), respectively, under its native promoter in the ΔBcCcp1 strain (Figure S3F). Inactivation of BcCcp1 had no effect on the vegetative growth but led to increased sensitivity to H_2O_2, and complementation with *BcCcp1* (ΔBcCcp1-C) completely restored this phenotype. The $BcCcp1^{H131L}$ and $BcCcp1^{K101A}$ mutants, which exhibited impaired enzymatic activity or secretion of this protein, were also more sensitive to H_2O_2 (Figure 3A). In addition, compared with the WT and the complement strains, >4-fold higher accumulation of H_2O_2 was observed after inoculation with the mutants ΔBcCcp1, $BcCcp1^{K101A}$, and $BcCcp1^{H131L}$ (Figure 3B). In support of these observations, injection of the WT BcCcp1-His6, but not the H131L mutant, into *Nicotiana benthamiana* leaves reduced H_2O_2 accumulation (Figure S4A) and cell death (Figure S4B) caused by BAX and, as a result, enhanced the infection of *Phytophthora capsici*, a biotrophic plant pathogen (Figure S4C).

To investigate whether ROS detoxification by BcCcp1 contributes to *B. cinerea* infection, a pathogenicity assay was carried out on mung bean leaves (Figure 3C). Among all the tested strains, ΔBcCcp1, $BcCcp1^{K101A}$, and $BcCcp1^{H131L}$ caused smaller lesions than B05.10 and the complement strain. Quantitative real-time PCR and western blot analyses showed that the transcript of *BcCcp1* (Figure 3D) was dramatically elevated, and the protein product (Figure 3E) was highly accumulated in the supernatant at 24 hpi and then decreased at 36 hpi, which was

Please cite this article in press as: Yang et al., Broad-spectrum chemicals block ROS detoxification to prevent plant fungal invasion, Current Biology (2022), https://doi.org/10.1016/j.cub.2022.07.022

similar to the expression pattern of BcTo11. These results suggest that *B. cinerea* secretes BcCcp1, which is modulated by BcTo11, to promote its infection through detoxification of ROS at the early infection stage.

Acetylation of BcTol1 by BcRtt109

To further examine the relation between acetylation of K122 and BcTol1 activity, we sought to identify the enzymes responsible for acetylation of this protein. Although *B. cinerea* contains multiple genes that may encode the putative acetyltranferase,[35] we initially focused on BcRtt109, a putative BcTol1 interacting protein identified in the coIP assay (Data S1). Rtt109 was defined as a histone acetyltransferase required for proper H3K56 acetylation in a previous study.[36] Our confocal microscopy and subcellular fractionation analyses revealed that although BcRtt109 was mainly localized in the nucleus, a small amount of this enzyme was also present in the cytosol (Figures 4A and 4B). CoIP assay proved the interaction between BcTol1 and BcRtt109 *in vivo* (Figure 4C). Accordingly, we determined the acetylation of BcTol1 in *BcRtt109* deletion and overexpression strains. Removal of BcRtt109 dramatically decreased acetylation of BcTol1 (Figure 4D), while overexpression of this enzyme increased acetylation of WT BcTol1 more than 2-fold, without obvious effect on the K122Q mutant protein (Figure 4E). To determine whether BcRtt109 can directly modify BcTol1, WT and mutant BcTol1-His6 proteins purified from bacterial cells were incubated with purified BcRtt109-GST or GST in the presence of acetyl-CoA. As shown in Figure 4F, purified BcRtt109-GST, but not GST itself, acetylates WT BcTol1 effectively *in vitro*, whereas it displays low activity against the K122Q mutant BcTol1 protein. Consistent with the acetylation level of BcTol1, the *BcRtt109* transcript and its protein product also decreased during the invasion process (Figures 4G and 4H). These data indicate that BcRtt109 is an enzyme that catalyzes K122 acetylation of BcTol1.

The amount of BcRtt109 reaches the maximum at 6 hpi, indicating that this enzyme is biologically important for *B. cinerea*. As expected, inactivation of BcRtt109 led to not only retarded growth but also dramatically decreased virulence of *B. cinerea*. As a negative regulator of BcTol1, overexpression of BcRtt109 slightly increased the growth rate but impaired the virulence of *B. cinerea* (Figures S5A and S5B). These results indicate that *B. cinerea* must tightly regulate the level of BcRtt109 to ensure its development and pathogenicity.

BcTol1 is a new drug target

Tight control of BcCcp1 secretion by BcTol1 enables it to be a promising target of fungicides. Through screening against the sub-database of ChemDiv, we identified 2 chemicals, 5664-0417 and 6623-1943, that putatively target the VHS domain of BcTol1 (Figure S6A). According to the interaction mode, 5664-0417 forms hydrophobic interaction with K76, R83, D120, and K122, and 6623-1943 forms hydrogen bond interaction with D120 and hydrophobic interaction with K76, R83, and K122 of BcTol1 (Figure 5A). Surface plasmon resonance (SPR) analysis showed that both 5664-0417 and 6623-1943 could bind the WT BcTol1 purified from *E. coli* cells, with the K_D values calculated by TraceDrawer of $4.22e^{-10}$ and $7.15e^{-9}$ mol/L, respectively (Figure 5B). However, after simultaneous mutation of the interacting residues (K76, R83, and D120 to A; K122 to Q), binding of these chemicals to the mutant proteins of BcTol1 is >14-fold weaker than binding to the WT protein (Figure 5C). Re-docking using the full structure further confirmed that 5664-0417 and 6623-1943 associate with K76, R83, D120, and K122, but not other regions of BcTol1 (Figure S6B). These results indicate that the two chemicals specifically target BcTol1.

We therefore examined whether these molecules can serve as anti-fungal chemicals to control *B. cinerea* infection. We found that application of 10 μM of these chemicals at the same time with *B. cinerea* spore inoculation resulted in up to 81% reduction of the disease lesions (Figure 5D). Application of 5664-0417 and 6623-1943 also prevents *B. cinerea* invasion of tomato fruits (Figure S6C). As shown in Figures 5E–5G, treatment with 5664-0417 or 6623-1943 completely disrupted the interaction of BcTol1 and BcCcp1 and, as a consequence, largely abolished BcCcp1 secretion and led to impaired antioxidant capacity of *B. cinerea*. Further dose gradient experiments ranging from 1 to 15 μM showed that treatment with 5.87 μM 5664-0417 or 5.03 μM 6623-1943 could reduce disease lesions by 50% (EC_{50}) (Figure 5H). No synergistic effect was observed when mixing the two chemicals in equal amounts to a final concentration of 10 μM (Figures 5D–5H), further supporting the notion that they share the same target. Moreover, application of 100 μM of these two chemicals on mung bean caused no visible growth defect (Figure S6D). Collectively, these results indicate that chemicals targeting BcTol1 to inhibit ROS detoxification are potentially effective fungicides to control gray mold.

Chemicals are effective against a broad spectrum of fungal pathogens

The chemical interacting residues of BcTol1 are conserved among multiple pathogenic fungi (Figure S1D), suggesting that the identified compounds might be effective in preventing invasion of these pathogens. To this end, three pathogenic fungi, *Magnaporthe oryzae*, *Fusarium graminearum*, and *Fusarium oxysporum*, were selected for further validation. As shown in Figure S6E, application of 5664-0417 and 6623-1943 at the same time with inoculation significantly decreased the severity of rice blast disease caused by *M. oryzae*, reducing the lesion length by 65% and 70%, respectively. Application of these chemicals at the same time with inoculation was also able to notably reduce the lesion size or disease index caused by *F. graminearum* and *F. oxysporum* on wheat coleoptiles and tomato seedlings, respectively (Figures S6F and S6G). Importantly, treatment with 100 μM of these small molecules had no obvious effect on rice, wheat, and tomato seedlings (Figures S6H–S6J). As oxidative stress tolerance is critical for pathogenicity of these fungi,[13,37,38] our results strongly suggest that chemicals blocking ROS detoxification could be effective fungicides to control crop diseases.

DISCUSSION

The studies presented here identify new chemicals that prevent infection by plant pathogenic fungi by blocking ROS detoxification. Specifically, we found that (1) BcTol1 is regulated at different levels to enhance its activity during the early infection stage of *B. cinerea*; (2) BcTol1 associates with ubiquitinated BcCcp1 through its VHS domain and modulates the secretion

Please cite this article in press as: Yang et al., Broad-spectrum chemicals block ROS detoxification to prevent plant fungal invasion, Current Biology (2022), https://doi.org/10.1016/j.cub.2022.07.022

CellPress
OPEN ACCESS

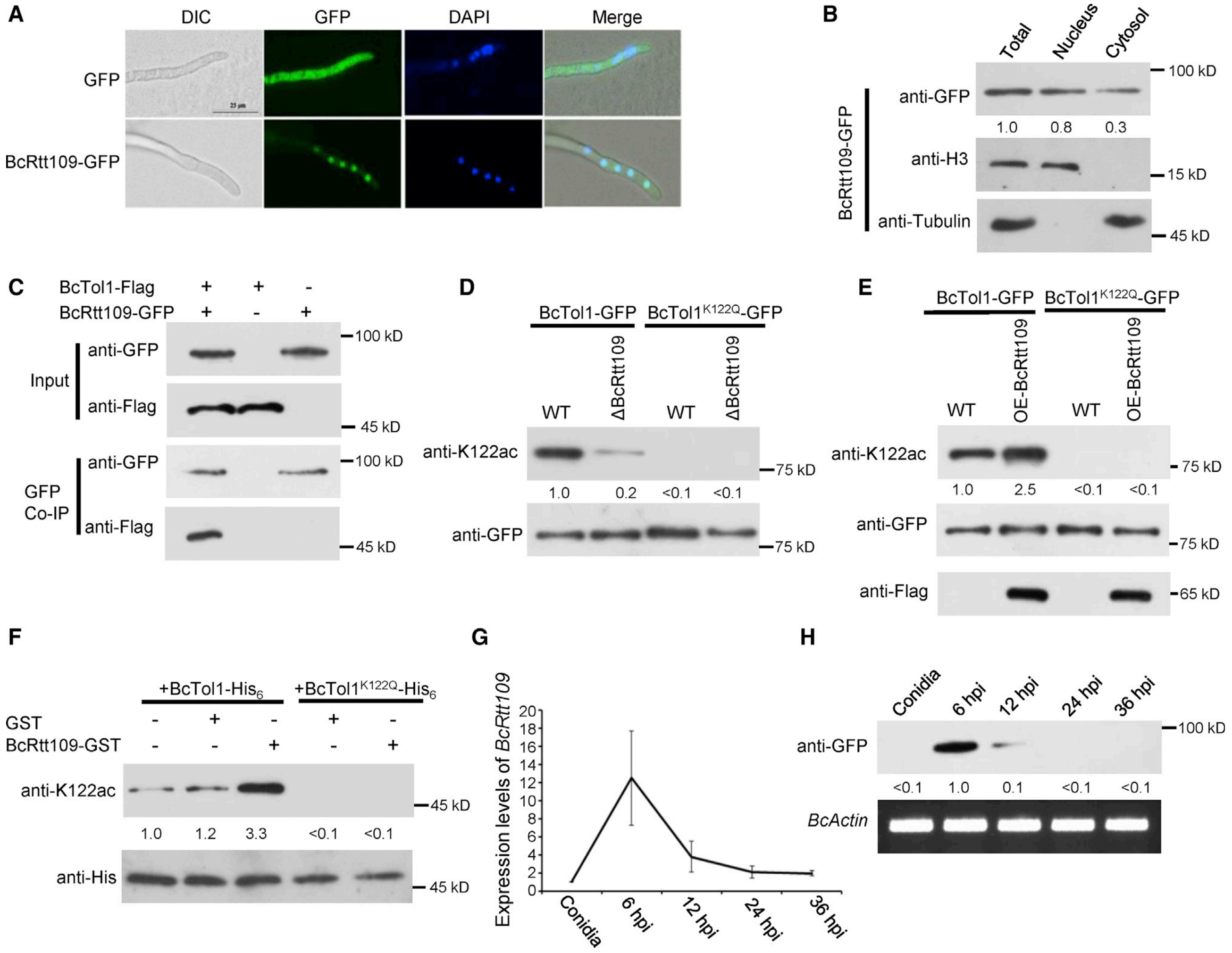

Figure 4. Acetylation of BcTol1 by BcRtt109

(A) Fluorescence microscopy analysis of BcRtt109-GFP localization. Scale bars, 25 μm.

(B) Subcellular fractionation of BcRtt109-GFP transformants in *B. cinerea*. Nuclear and cytoplasmic proteins were separately extracted, and BcRtt109-GFP was detected with anti-GFP antibody. The fractionation controls were histone H3 (nucleus) and tubulin (cytosol). The amount of total BcRtt109 was set at 1.

(C) Association of BcTol1 with BcRtt109 *in vivo*. BcTol1 fused with FLAG and BcRtt109 fused with GFP were cotransformed into *B. cinerea*. Proteins pulled down with GFP-Trap beads were probed with anti-FLAG and anti-GFP antibodies.

(D) The K122 acetylation (top) and amount (bottom) of BcTol1-GFP and BcTol1^{K122Q}-GFP in B05.10 and ΔBcRtt109 strains.

(E) The K122 acetylation (top) and amount (bottom) of BcTol1-GFP and BcTol1^{K122Q}-GFP in B05.10 and *BcRtt109* overexpression strains.

For (D) and (E), proteins pulled down with GFP-Trap beads were probed with anti-K122ac, anti-GFP, and anti-FLAG antibodies. The amount of acetylated WT BcTol1 in B05.10 was set at 1.

(F) BcRtt109 directly acetylates BcTol1 *in vitro*. Purified BcTol1-His6 or BcTol1^{K122Q}-His6 (10 μg) was incubated with 10 μg of purified BcRtt109-GST or GST in the presence of 0.2 mM acetyl-CoA and then analyzed by immunoblotting using anti-K122ac or anti-His antibody. The amount of acetylated BcTol1 without addition of BcRtt109-GST or GST was set at 1.

(G) Relative level of *BcRtt109* transcript during the infection stage.

(H) Amount of BcRtt109 during the infection stage. *B. cinerea Actin* gene was used as the loading control. The amount of BcRtt109 at 6 hpi was set as 1.

See also Figure S5.

of this peroxidase; (3) secreted BcCcp1 eliminates plant-produced H_2O_2 to promote *B. cinerea* infection; (4) inactivation of BcTol1 or BcCcp1 intervenes in ROS scavenging and impairs virulence of *B. cinerea*; (5) two small molecules targeting the VHS domain of BcTol1 block the secretion of BcCcp1, ultimately leading to effective prevention of *B. cinerea* invasion; and (6) the chemicals display effectiveness against a broad spectrum of plant fungal pathogens. Although BcTol1 may also be involved in other biological processes, these findings support a model in which BcTol1 regulates pathogenicity of *B. cinerea* mainly through modulating BcCcp1 secretion-dependent ROS detoxification (Figure 6). Therefore, BcTol1 can serve as a new drug target. The identification of chemicals inhibiting peroxidase secretion that consequently prevents ROS-scavenging-mediated plant infection therefore offers an effective strategy for controlling fungal diseases.

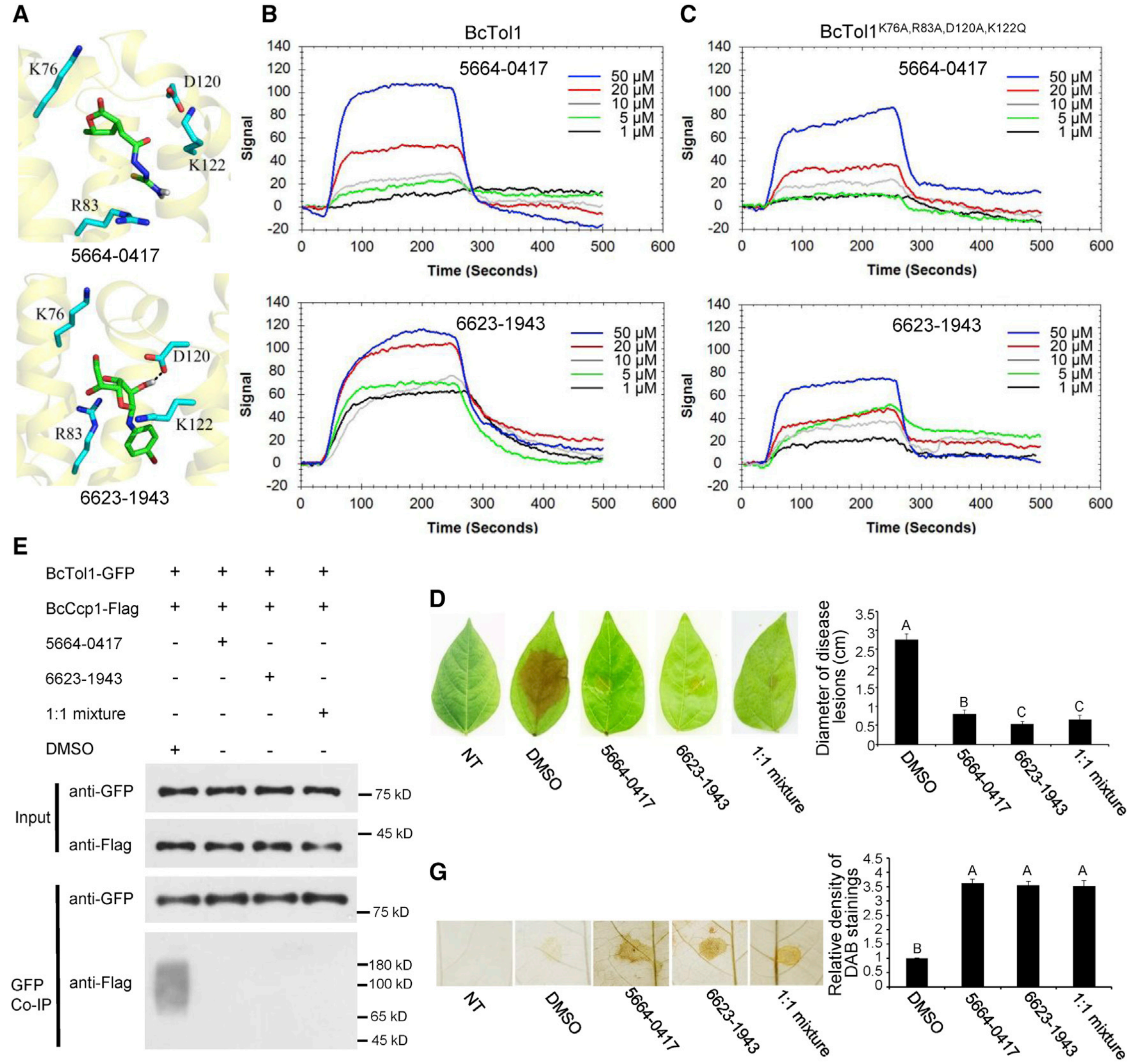

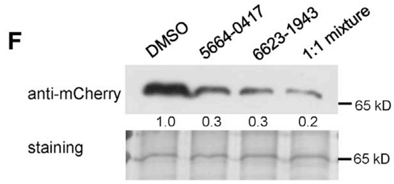

H

Chemical	5664-0417	6623-1943	1:1 mixture
EC_{50}	5.87±0.84	5.03±0.46	5.10±0.10

Figure 5. Inhibitor effect of BcTol1 targeting chemicals on *B. cinerea* invasion and ROS detoxification

(A) Molecular docking model of 5664-0417, 6623-1943, and BcTol1.

(B) SPR analysis of 5664-0417 and 6623-1943 binding to WT BcTol1.

(C) SPR analysis of 5664-0417 and 6623-1943 binding to mutant BcTol1 proteins.

(D) Virulence of *B. cinerea* on mung bean leaves with or without the application of 5664-0417 and 6623-1943. The chemicals were mixed with conidia suspension to a final concentration of 10 μM, and inoculation was then performed.

(E) Association of BcTol1 with BcCcp1 *in vivo* with or without the application of 5664-0417 and 6623-1943. After treatment with DMSO or 10 μM 5664-0417, 6623-1943, or their mixture (1:1) for 3 h, total proteins were extracted from the mycelia of BcTol1-GFP and BcCcp1-FLAG carrying strain. CoIP and western blot analyses were then performed as in Figure 2.

(F) Secretion of BcCcp1 with or without the application of 5664-0417 and 6623-1943. Total proteins were extracted from culture supernatant after inoculation for 24 h in the presence of DMSO or 10 μM 5664-0417, 6623-1943, or their mixture (1:1) and probed with anti-mCherry antibody as in Figure 2. The amount of BcCcp1 treated with DMSO was set as 1. Silver staining shows protein loading to each lane.

(G) DAB staining shows ROS accumulation in mung bean leaves after infection by B05.10 with or without the application of 5664-0417 and 6623-1943.

(H) EC_{50} values of 5664-0417, 6623-1943, and their mixture (1:1) for disease lesions of *B. cinerea*.

See also Figure S6.

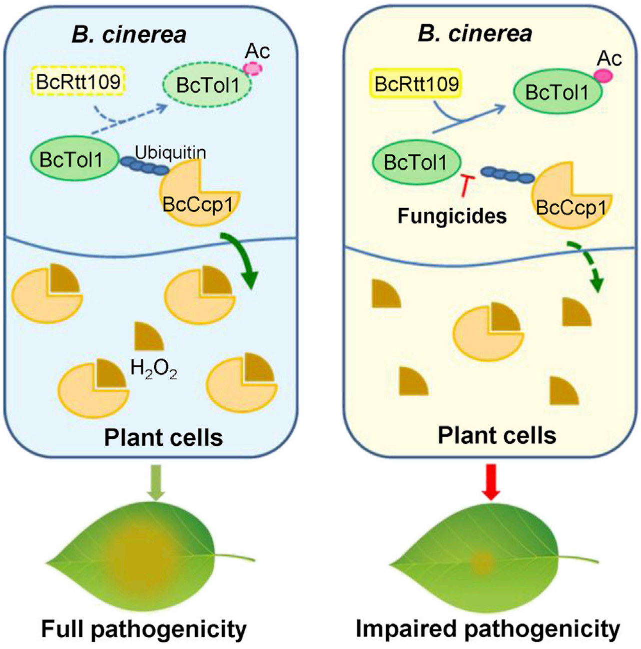

Figure 6. A model for the role of BcTol1 in BcCcp1-mediated ROS detoxification and pathogenicity of *B. cinerea*

During the early infection stage, *B. cinerea* downregulates BcRtt109 to reduce BcTol1 acetylation, enabling its association with ubiquitinated BcCcp1, thereby increasing the secretion of this enzyme. Secreted BcCcp1 eliminates plant-produced H_2O_2, leading to invasion of *B. cinerea*. Disruption of the interaction of BcTol1 with BcCcp1 by either inhibitors or acetylation of BcTol1 by BcRtt109 blocks BcCcp1 secretion, preventing ROS detoxification, and as a consequence, *B. cinerea* fails to infect the host plant.

See also Figure S6.

Tom1 family proteins are major components of the protein trafficking system. Therefore, cells must elaborately control their activities to ensure correct delivery of functionally important protein molecules. In mammalian cells, the expression of Tom1 is significantly induced by oncogenic MYB1.[39] Arabidopsis Tol6 is ubiquitinated *in planta*, and this modification affects the regulation of cargo sorting via spatiotemporal control of subcellular Tol distribution.[23] Our results indicate not only that BcTol1 is upregulated at transcriptional and translational levels, but its acetylation, catalyzed by the lysine acetyltransferase BcRtt109, decreases during the early infection stage of *B. cinerea* (Figures 1A–1C). The dual regulation of BcTol1 ultimately leads to enhanced activity of BcTol1 and thus secretion of BcCcp1 and consequently promotes infection of the pathogen. All these findings indicate that cells employ a variety of mechanisms to carefully control the amount, activity, specificity, and cellular localization of Tom1 family proteins. This complex regulatory system serves as a salient example of how eukaryotes can control their development through regulating the action of a cargo sorting protein.

The production of ROS is a ubiquitous defense response in plants, and pathogens would have evolved numerous mechanisms to counteract this deleterious effect. One key approach is enzymatic ROS scavenging through the secretion of antioxidant enzymes.[40] Ccp, which efficiently couples H_2O_2 to the one-electron oxidation of two ferrocytochrome *c* molecules, is capable of inactivating ROS in the host.[41] However, until now, the function of Ccp in plant pathogens has been poorly characterized. In this research, we found that secretion of BcCcp1 by *B. cinerea* during the early infection stage reduces H_2O_2 accumulation by >80% in plants and thus facilitates pathogen invasion (Figure 3B). In support of our observations, two Ccp genes of *M. oryzae* were upregulated at 78 hpi and deletion of one of them, *MoCcp1*, led to decreased pathogenicity on rice.[16] Therefore, ROS detoxification is likely critical for the pathogenicity of not only biotrophs, but also necrotrophic pathogens, and inhibitors of this process might be used as fungicides for controlling of plant diseases.

Currently, the main method of plant disease control is application of agrochemicals.[4] For the currently used fungicides, their targets are all essential housekeeping genes. For example, the fungicides benzimidazole, demethylation inhibitors, succinate dehydrogenase inhibitors, and quinone outside inhibitors target β-tubulin assembly, ergosterol biosynthesis, succinate dehydrogenase, and cytochrome *b*, respectively.[42–45] Due to the global concern[46] of drug resistance and off-target effects, fungicides targeting pathogenicity-specific genes are urgently required in disease control. In this study, we have illustrated that two small molecules targeting BcTol1 are effective inhibitors of ROS detoxification (Figure 5). These chemicals are effective against plant fungal pathogens without an obvious side effect on the host plants (Figures 5 and S6). Importantly, the chemical interacting residues are absent in mammalian Tom1 and Tol proteins (Figure S1D), suggesting that these small molecules are safe for humans. The compounds could thus be employed as broad-spectrum drugs targeting a conserved mechanism in which ROS detoxification requires Tom1/Tols-dependent secretion of antioxidant enzymes as in many fungal pathogens. The approach of identifying BcTol1 inhibitors has wide implications for further fungicide discovery.

The potential resistance risk of the new fungicides is still high, including 5664-0417 and 6623-1943 developed in this study. Meanwhile, the time from finding a suitable target gene to commercial products is quite long, with many difficulties likely to be involved.[47] Site-specific fungicides are important for the immediate problems faced in efforts to protect, but they are not a sustainable solution unless integrated into a program of management in administration including genetic and cultural control.[48,49]

STAR★METHODS

Detailed methods are provided in the online version of this paper and include the following:

- KEY RESOURCES TABLE
- RESOURCE AVAILABILITY
  - Lead contact
  - Materials availability
  - Data and code availability
- EXPERIMENTAL MODEL AND SUBJECT DETAILS
- METHOD DETAILS
  - Construction of gene deletion, complementation, site-directed mutagenesis, and GFP, Flag and mCherry fusion vectors
  - RNA extraction and quantitative reverse transcription PCR (qRT-PCR)

- ○ Yeast two-hybrid assays
- ○ Co-immunoprecipitation (Co-IP) assays
- ○ Mass spectrometry analysis
- ○ Generation of anti-K122ac-BcTol1 and anti-K101ubq-BcCcp1 antibodies
- ○ Western blot analysis
- ○ Peroxidase activity measurement
- ○ Plant cultivation conditions
- ○ Pathogenicity and infection-related morphogenesis assays
- ○ ROS detoxification of BcCcp1 in *Nicotiana benthamiana*
- ○ Fluorescence microscopy
- ○ Subcellular fractionation analysis
- ○ Lysine acetylation reaction assay *in vitro*
- ○ Molecular docking analysis
- ○ Surface plasmon resonance (SPR) analysis
- ● QUANTIFICATION AND STATISTICAL ANALYSIS

SUPPLEMENTAL INFORMATION

Supplemental information can be found online at https://doi.org/10.1016/j.cub.2022.07.022.

ACKNOWLEDGMENTS

This research was supported by the Shandong Provincial Natural Science Foundation (ZR2020KC003), the Shandong Province ‘‘Double-Hundred Talent Plan’’ (WST2018008), the National Natural Science Foundation of China (31972213), and the Taishan Scholar Construction Foundation of Shandong Province (tshw20130963). J.Y. was supported by the Major Tobacco Green Prevention and Control Project 110201901041(ls-04). B.F.L. was supported by the Wellcome Trust Investigator awards (200873/Z/16/Z and 222451/Z/21/Z). We are grateful to Professor Xiaojun Yao for providing his Schrödinger software packages.

AUTHOR CONTRIBUTIONS

Q.Y. and W.L. generated the hypothesis and planned the experiments. Q.Y., J.Y., Y.W., J.D., and J.Z. performed the experiments. B.F.L. provided technical support. Q.Y., W.L., and B.F.L. wrote the paper.

DECLARATION OF INTERESTS

Q.Y., Y.W., J.Z., and W.L. are associated with a provisional patent, BcTol1-based inhibitors of plant fungal pathogens (202210316770.1), related to the work.

Received: April 19, 2022
Revised: June 16, 2022
Accepted: July 12, 2022
Published: August 5, 2022

REFERENCES

1. Fisher, M.C., Henk, D.A., Briggs, C.J., Brownstein, J.S., Madoff, L.C., McCraw, S.L., and Gurr, S.J. (2012). Emerging fungal threats to animal, plant and ecosystem health. Nature *484*, 186–194.
2. Strange, R.N., and Scott, P.R. (2005). Plant disease: a threat to global food security. Annu. Rev. Phytopathol. *43*, 83–116.
3. Ma, Z., and Michailides, T.J. (2005). Advances in understanding molecular mechanisms of fungicide resistance and molecular detection of resistant genotypes in phytopathogenic fungi. Crop Prot. *24*, 853–863.
4. Fisher, M.C., Hawkins, N.J., Sanglard, D., and Gurr, S.J. (2018). Worldwide emergence of resistance to antifungal drugs challenges human health and food security. Science *360*, 739–742.
5. Kaczyński, P., Łozowicka, B., Perkowski, M., Zoń, W., Hrynko, I., Rutkowska, E., and Skibko, Z. (2021). Impact of broad-spectrum pesticides used in the agricultural and forestry sector on the pesticide profile in wild boar, roe deer and deer and risk assessment for venison consumers. Sci. Total Environ. *784*, 147215.
6. Syafrudin, M., Kristanti, R.A., Yuniarto, A., Hadibarata, T., Rhee, J., Al-Onazi, W.A., Algarni, T.S., Almarri, A.H., and Al-Mohaimeed, A.M. (2021). Pesticides in drinking water-a review. Int. J. Environ. Res. Public Health *18*, 468.
7. He, M., Su, J., Xu, Y., Chen, J., Chern, M., Lei, M., Qi, T., Wang, Z., Ryder, L.S., Tang, B., et al. (2020). Discovery of broad-spectrum fungicides that block septin-dependent infection processes of pathogenic fungi. Nat. Microbiol. *5*, 1565–1575.
8. Zhao, B., Fan, S., Fan, Z., Wang, H., Zhang, N., Guo, X., Yang, D., Wu, Q., Yu, B., and Zhou, S. (2018). Discovery of pyruvate kinase as a novel target of new fungicide candidate 3-(4-methyl-1, 2, 3-thiadiazolyl)-6-trichloromethyl-[1, 2, 4]-triazolo-[3, 4- b] [1, 3, 4]-thiadizole. J. Agric. Food Chem. *66*, 12439–12452.
9. Yoshioka, H., Bouteau, F., and Kawano, T. (2008). Discovery of oxidative burst in the field of plant immunity: looking back at the early pioneering works and towards the future development. Plant Signal. Behav. *3*, 153–155.
10. O'Brien, J.A., Daudi, A., Butt, V.S., and Bolwell, G.P. (2012). Reactive oxygen species and their role in plant defence and cell wall metabolism. Planta *236*, 765–779.
11. Mur, L.A., Kenton, P., Lloyd, A.J., Ougham, H., and Prats, E. (2008). The hypersensitive response; the centenary is upon us but how much do we know? J. Exp. Bot. *59*, 501–520.
12. Wang, N., Fan, X., He, M., Hu, Z., Tang, C., Zhang, S., Lin, D., Gan, P., Wang, J., Huang, X., et al. (2022). Transcriptional repression of TaNOX10 by TaWRKY19 compromises ROS generation and enhances wheat susceptibility to stripe rust. Plant Cell *34*, 1784–1803.
13. Lee, Y., Son, H., Shin, J.Y., Choi, G.J., and Lee, Y.W. (2018). Genome-wide functional characterization of putative peroxidases in the head blight fungus *Fusarium graminearum*. Mol. Plant Pathol. *19*, 715–730.
14. Huang, K., Czymmek, K.J., Caplan, J.L., Sweigard, J.A., and Donofrio, N.M. (2011). HYR1-mediated detoxification of reactive oxygen species is required for full virulence in the rice blast fungus. PLoS Pathog *7*, e1001335.
15. Missall, T.A., Pusateri, M.E., and Lodge, J.K. (2004). Thiol peroxidase is critical for virulence and resistance to nitric oxide and peroxide in the fungal pathogen. *Cryptococcus neoformans*. Mol. Microbiol. *51*, 1447–1458.
16. Mir, A.A., Park, S.Y., Abu Sadat, M., Kim, S., Choi, J., Jeon, J., and Lee, Y.H. (2015). Systematic characterization of the peroxidase gene family provides new insights into fungal pathogenicity in *Magnaporthe oryzae*. Sci. Rep. *5*, 11831.
17. Yang, S.L., Yu, P.L., and Chung, K.R. (2016). The glutathione peroxidase-mediated reactive oxygen species resistance, fungicide sensitivity and cell wall construction in the citrus fungal pathogen *Alternaria alternata*. Environ. Microbiol. *18*, 923–935.
18. Mercier, A., Simon, A., Lapalu, N., Giraud, T., Bardin, M., Walker, A.S., Viaud, M., and Gladieux, P. (2021). Population genomics reveals molecular determinants of specialization to tomato in the polyphagous fungal pathogen *Botrytis cinerea* in France. Phytopathology *111*, 2355–2366.
19. Gil-Ad, N.L., Bar-Nun, N., Noy, T., and Mayer, A.M. (2000). Enzymes of *Botrytis cinerea* capable of breaking down hydrogen peroxide. FEMS Microbiol. Lett. *190*, 121–126.
20. Dell'Angelica, E.C., Puertollano, R., Mullins, C., Aguilar, R.C., Vargas, J.D., Hartnell, L.M., and Bonifacino, J.S. (2000). GGAs: a family of ADP

ribosylation factor-binding proteins related to adaptors and associated with the Golgi complex. J. Cell Biol. *149*, 81–94.

21. Lohi, O., and Lehto, V.P. (1998). VHS domain marks a group of proteins involved in endocytosis and vesicular trafficking. FEBS Lett *440*, 255–257.

22. Korbei, B., Moulinier-Anzola, J., De-Araujo, L., Lucyshyn, D., Retzer, K., Khan, M.A., and Luschnig, C. (2013). Arabidopsis TOL proteins act as gatekeepers for vacuolar sorting of PIN2 plasma membrane protein. Curr. Biol. *23*, 2500–2505.

23. Moulinier-Anzola, J., Schwihla, M., De-Araújo, L., Artner, C., Jörg, L., Konstantinova, N., Luschnig, C., and Korbei, B. (2020). TOLs function as ubiquitin receptors in the early steps of the ESCRT pathway in higher plants. Mol. Plant *13*, 717–731.

24. Roach, T.G., Lång, H.K.M., Xiong, W., Ryhänen, S.J., and Capelluto, D.G.S. (2021). Protein trafficking or cell signaling: a dilemma for the adaptor protein TOM1. Front. Cell Dev. Biol. *9*, 643769.

25. Boal, F., Mansour, R., Gayral, M., Saland, E., Chicanne, G., Xuereb, J.M., Marcellin, M., Burlet-Schiltz, O., Sansonetti, P.J., Payrastre, B., and Tronchère, H. (2015). TOM1 is a PI5P effector involved in the regulation of endosomal maturation. J. Cell Sci. *128*, 815–827.

26. Williamson, B., Tudzynski, B., Tudzynski, P., and van Kan, J.A. (2007). *Botrytis cinerea*: the cause of grey mould disease. Mol. Plant Pathol. *8*, 561–580.

27. Dean, R., Van Kan, J.A., Pretorius, Z.A., Hammond-Kosack, K.E., Di Pietro, A., Spanu, P.D., Rudd, J.J., Dickman, M., Kahmann, R., Ellis, J., and Foster, G.D. (2012). The top 10 fungal pathogens in molecular plant pathology. Mol. Plant Pathol. *13*, 414–430.

28. Fillinger, S., and Elad, Y. (2016). *Botrytis* – the Fungus, the Pathogen and Its Management in Agricultural Systems (Springer International Publishing), pp. 413–486.

29. Lv, B., Yang, Q., Li, D., Liang, W., and Song, L. (2016). Proteome-wide analysis of lysine acetylation in the plant pathogen Botrytis cinerea. Sci. Rep. *6*, 29313.

30. Li, X., Zhang, S., Blander, G., Tse, J.G., Krieger, M., and Guarente, L. (2007). SIRT1 deacetylates and positively regulates the nuclear receptor LXR. Mol. Cell *28*, 91–106.

31. Schwer, B., Bunkenborg, J., Verdin, R.O., Andersen, J.S., and Verdin, E. (2006). Reversible lysine acetylation controls the activity of the mitochondrial enzyme acetyl-CoA synthetase 2. Proc. Natl. Acad. Sci. USA *103*, 10224–10229.

32. Chi, M.H., Park, S.Y., Kim, S., and Lee, Y.H. (2009). A novel pathogenicity gene is required in the rice blast fungus to suppress the basal defenses of the host. PLoS Pathog *5*, e1000401.

33. Gold, M.H., Kuwahara, M., Chiu, A.A., and Glenn, J.K. (1984). Purification and characterization of an extracellular H2O2-requiring diarylpropane oxygenase from the white rot basidiomycete, *Phanerochaete chrysosporium*. Arch. Biochem. Biophys. *234*, 353–362.

34. Zhu, W., Ronen, M., Gur, Y., Minz-Dub, A., Masrati, G., Ben-Tal, N., Savidor, A., Sharon, I., Eizner, E., Valerius, O., et al. (2017). BcXYG1, a secreted xyloglucanase from *Botrytis cinerea*, triggers both cell death and plant immune responses. Plant Physiol *175*, 438–456.

35. Van Kan, J.A., Stassen, J.H., Mosbach, A., Van Der Lee, T.A., Faino, L., Farmer, A.D., Papasotiriou, D.G., Zhou, S., Seidl, M.F., Cottam, E., et al. (2017). A gapless genome sequence of the fungus *Botrytis cinerea*. Mol. Plant Pathol. *18*, 75–89.

36. Schneider, J., Bajwa, P., Johnson, F.C., Bhaumik, S.R., and Shilatifard, A. (2006). Rtt109 is required for proper H3K56 acetylation: a chromatin mark associated with the elongating RNA polymerase II. J. Biol. Chem. *281*, 37270–37274.

37. Tanabe, S., Nishizawa, Y., and Minami, E. (2010). Effects of catalase on the accumulation of H2O2 in rice cells inoculated with rice blast fungus, *Magnaporthe oryzae*. Physiol. Plant. *137*, 148–154.

38. Manzo, D., Ferriello, F., Puopolo, G., Zoina, A., D'Esposito, D., Tardella, L., Ferrarini, A., and Ercolano, M.R. (2016). *Fusarium oxysporum f.sp. radicis-lycopersici* induces distinct transcriptome reprogramming in resistant and susceptible isogenic tomato lines. BMC Plant Biol *16*, 53.

39. Burk, O., Worpenberg, S., Haenig, B., and Klempnauer, K.H. (1997). Tom-1, a novel v-Myb target gene expressed in AMV- and E26-transformed myelomonocytic cells. EMBO J *16*, 1371–1380.

40. Belozerskaya, T.A., and Gessler, N.N. (2007). Reactive oxygen species and the strategy of antioxidant defense in fungi: a review. Appl. Biochem. Microbiol. *43*, 506–515.

41. Volkov, A.N., Nicholls, P., and Worrall, J.A. (2011). The complex of cytochrome c and cytochrome c peroxidase: the end of the road? Biochim. Biophys. Acta *1807*, 1482–1503.

42. Hollomon, D.W., Butters, J.A., Barker, H., and Hall, L. (1998). Fungal beta-tubulin, expressed as a fusion protein, binds benzimidazole and phenylcarbamate fungicides. Antimicrob. Agents Chemother. *42*, 2171–2173.

43. Li, S., Li, X., Zhang, H., Wang, Z., and Xu, H. (2021). The research progress in and perspective of potential fungicides: succinate dehydrogenase inhibitors. Bioorg. Med. Chem. *50*, 116476.

44. Yin, D., Chen, X., Hamada, M.S., Yu, M., Yin, Y., and Ma, Z. (2015). Multiple resistance to QoIs and other classes of fungicides in *Botrytis cinerea* populations from strawberry in Zhejiang Province, China. Eur. J. Plant Pathol. *141*, 169–177.

45. Shao, W., Zhao, Y., and Ma, Z. (2021). Advances in understanding fungicide resistance in *Botrytis cinerea* in China. Phytopathology *111*, 455–463.

46. Leroux, P. (2007). Chemical Control of *Botrytis* and Its Resistance to Chemical Fungicides (Springer Netherlands).

47. Jampilek, J. (2016). Potential of agricultural fungicides for antifungal drug discovery. Expert Opin. Drug Discov. *11*, 1–9.

48. Kokhmetova, A., Rsaliyev, A., Malysheva, A., Atishova, M., Kumarbayeva, M., and Keishilov, Z. (2021). Identification of stripe rust resistance genes in common wheat cultivars and breeding lines from Kazakhstan. Plants (Basel) *10*, 2303.

49. Peshin, R., Bandral, R.S., Zhang, W.J., Wilson, L., and Dhawan, A.K. (2009). Integrated pest management: a global overview of history, programs and adoption. In Integrated Pest Management: Innovation-Development Process (Springer), pp. 1–49.

50. Yang, Q., Zhang, J., Hu, J., Wang, X., Lv, B., and Liang, W. (2018). Involvement of BcYak1 in the regulation of vegetative differentiation and adaptation to oxidative stress of *Botrytis cinerea*. Front. Microbiol. *9*, 281.

51. Schumacher, J. (2012). Tools for *Botrytis cinerea*: new expression vectors make the gray mold fungus more accessible to cell biology approaches. Fungal Genet. Biol. *49*, 483–497.

52. Sali, A. (1995). Comparative protein modeling by satisfaction of spatial restraints. Mol. Med. Today *1*, 270–277.

53. Sastry, G.M., Adzhigirey, M., Day, T., Annabhimoju, R., and Sherman, W. (2013). Protein and ligand preparation: parameters, protocols, and influence on virtual screening enrichments. J. Comput. Aided Mol. Des. *27*, 221–234.

54. Quidde, T., Büttner, P., and Tudzynski, P. (1999). Evidence for three different specific saponin-detoxifying activities in *Botrytis cinerea* and cloning and functional analysis of a gene coding for a putative avenacinase. Eur. J. Plant Pathol. *105*, 273–283.

55. Yu, J.H., Hamari, Z., Han, K.H., Seo, J.A., Reyes-Domínguez, Y., and Scazzocchio, C. (2004). Double-joint PCR: a PCR-based molecular tool for gene manipulations in filamentous fungi. Fungal Genet. Biol. *41*, 973–981.

56. Gronover, C.S., Kasulke, D., Tudzynski, P., and Tudzynski, B. (2001). The role of G protein alpha subunits in the infection process of the gray mold fungus *Botrytis cinerea*. Mol. Plant Microbe Interact. *14*, 1293–1302.

57. Livak, K.J., and Schmittgen, T.D. (2001). Analysis of relative gene expression data using real-time quantitative PCR and the $2^{-\Delta\Delta CT}$ method. Methods *25*, 402–408.

58. Yun, Y., Liu, Z., Zhang, J., Shim, W.B., Chen, Y., and Ma, Z. (2013). The MAPKK FgMkk1 of *Fusarium graminearum* regulates vegetative differentiation, multiple stress response, and virulence via the cell wall integrity and high-osmolarity glycerol signaling pathways. Environ. Microbiol. *16*, 2023–2037.

Please cite this article in press as: Yang et al., Broad-spectrum chemicals block ROS detoxification to prevent plant fungal invasion, Current Biology (2022), https://doi.org/10.1016/j.cub.2022.07.022

59. Schiestl, R.H., and Gietz, R.D. (1989). High efficiency transformation of intact yeast cells using single stranded nucleic acids as a carrier. Curr. Genet. *16*, 339–346.

60. Zhang, N., Song, L., Xu, Y., Pei, X., Luisi, B.F., and Liang, W. (2021). The decrotonylase FoSir5 facilitates mitochondrial metabolic state switching in conidial germination of *Fusarium oxysporum*. eLife *10*, e75583.

61. Liu, X., Zhou, Q., Guo, Z., Liu, P., Shen, L., Chai, N., Qian, B., Cai, Y., Wang, W., Yin, Z., et al. (2020). A self-balancing circuit centered on MoOsm1 kinase governs adaptive responses to host-derived ROS in *Magnaporthe oryzae*. eLife *9*, e61605.

62. Ren, X., and Hurley, J.H. (2010). VHS domains of ESCRT-0 cooperate in high-avidity binding to polyubiquitinated cargo. EMBO J *29*, 1045–1054.

Please cite this article in press as: Yang et al., Broad-spectrum chemicals block ROS detoxification to prevent plant fungal invasion, Current Biology (2022), https://doi.org/10.1016/j.cub.2022.07.022

STAR★METHODS

KEY RESOURCES TABLE

REAGENT or RESOURCE	SOURCE	IDENTIFIER
Antibodies		
anti-GFP antibody	Abcam	Cat# ab183734; RRID:AB_2732027
anti-mCherry antibody	Abcam	Cat# ab183628; RRID:AB_2650480
anti-Flag antibody	Sigma	Cat# F1804; RRID:AB_262044
anti-K122ac antibody	This paper	N/A
anti-K101ubq antibody	This paper	N/A
anti-His_6 antibody	TransGen Biotech	HT501-01
anti-GST antibody	ABclonal Technology	AE001
anti-H3 antibody	Abcam	Cat# ab1791; RRID:AB_302613
anti-tubulin antibody	PTM Biolabs	PTM-1011
Bacterial and virus strains		
Escherichia coli BL21 (DE3)	Invitrogen	C600003
E. coli DH5α	Invitrogen	18265017
Biological samples		
Mung bean tissue (*Vigna radiata*)	N/A	N/A
Tomato tissue (*Lycopersicon esculentum*)	N/A	N/A
Wheat tissue (*Triticum aestivum L.*)	N/A	N/A
Rice tissue (*Oryza sativa L.*)	N/A	N/A
Tobacoo tissue (*Nicotiana benthamiana*)	N/A	N/A
Chemicals, peptides, and recombinant proteins		
anti-GFP agarose	KT Health	KTSM1301
Hygromycin B	Thermo Fisher Scientific	10687010
G418	Solarbio	IG0010
DAPI	Solarbio	C0065
Proteinase inhibitor cocktail	Roche	5892791001
Critical commercial assays		
Pro Ligation-Free Cloning Kit	ABM	E086
Nuclear Protein Extraction Kit	Solarbio	R0050
All-In-One RT MasterMix	ABM	G492
SYBR Premix Ex Taq	Takara	RR420
Experimental models: Organisms/strains		
Botrytis cinerea	N/A	B05.10
Magnaporthe oryzae	N/A	Guy11
Fusarium graminearum	N/A	PH-1
Fusarium oxysporum f. sp. *Lycopersici*	N/A	Fo4287
ΔBcTol1	This study	ΔBcTol1
ΔBcTol1-C	This study	ΔBcTol1; *BcTol1*
BcTol1^{K122Q}	This study	ΔBcTol1; *BcTol1*^{K122Q}
BcTol1^{K122R}	This study	ΔBcTol1; *BcTol1*^{K122R}
BcTol1-GFP/BcCcp1-Flag	This study	*BcTol1-gfp*; olic:*BcCcp1-flag*
BcTol1^{K122Q}-GFP/BcCcp1-Flag	This study	BcTol1^{K122Q}*-gfp*; olic:*BcCcp1-flag*
BcTol1^{K122R}-GFP / BcCcp1-Flag	This study	*BcTol1*^{K122R}*-gfp*; olic:*BcCcp1-flag*
BcTol1$^{\Delta VHS}$-GFP/BcCcp1-Flag	This study	*BcTol1*$^{\Delta VHS}$*-gfp*; olic:*BcCcp1-flag*
BcTol1-GFP/BcCcp1^{K101A}-Flag	This study	*BcTol1-gfp*; olic:*BcCcp1*^{K101A}*-flag*
BcTol1-GFP/BcCcp1^{H131L}-Flag	This study	*BcTol1-gfp*; olic:*BcCcp1*^{H131L}*-flag*

(*Continued on next page*)

Please cite this article in press as: Yang et al., Broad-spectrum chemicals block ROS detoxification to prevent plant fungal invasion, Current Biology (2022), https://doi.org/10.1016/j.cub.2022.07.022

Continued

REAGENT or RESOURCE	SOURCE	IDENTIFIER
ΔBcCcp1	This study	ΔBcCcp1
ΔBcCcp1-C	This study	ΔBcCcp1; *BcCcp1*
BcCcp1^{H131L}	This study	ΔBcCcp1; *BcCcp1*^{H131L}
BcCcp1^{K101A}	This study	ΔBcCcp1; *BcCcp1*^{K101A}
BcCcp1-mCherry	This study	ΔBcCcp1; *BcCcp1-mCherry*
ΔBcRtt109	This study	ΔBcRtt109
OE-BcRtt109	This study	olic:*BcRtt109-flag*
BcRtt109-GFP	This study	ΔBcRtt109; *BcRtt109- gfp*
BcTol1-Flag/BcRtt109-GFP	This study	olic:*BcTol1-flag*; *BcRtt109-gfp*
ΔBcRtt109/BcTol1-GFP	This study	ΔBcRtt109; *BcTol1-gfp*
ΔBcRtt109/BcTol1^{K122Q}-GFP	This study	ΔBcRtt109; *BcTol1*^{K122Q}*- gfp*
OE-BcRtt109/BcTol1-GFP	This study	olic:*BcRtt109-flag*; *BcTol1-gfp*
OE-BcRtt109/BcTol1^{K122Q}-GFP	This study	olic:*BcRtt109-flag*; *BcTol1*^{K122Q}*-gfp*
**Oligonucleotides**		
Primers	Table S1	N/A
**Recombinant DNA**		
pBS-neo	Yang et al.[50]	N/A
pNAN-OGG	Schumacher[51]	N/A
pNAB-OCT	Schumacher[51]	N/A
phz126-olicP	This paper	N/A
pGBKT7	Clontech	630489
pGADT7	Clontech	K1612-1
pET-28a(+)	Sangon Biotech	B540183
pGEX-4T-2	GE Healthcare	27-4581-01
BcTol1-5'-HPH-3'	N/A	ΔBcTol1
BcTol1-pBS-neo	pBS-neo	ΔBcTol1-C
BcTol1^{K122Q}-pBS-neo	pBS-neo	BcTol1^{K122Q}
BcTol1^{K122R}-pBS-neo	pBS-neo	BcTol1^{K122R}
BcTol1-GFP	pNAN-OGG	BcTol1-GFP
BcCcp1-Flag	phz126-olicP-Flag	BcCcp1-Flag
BcTol1^{K122Q}-GFP	pNAN-OGG	BcTol1^{K122Q}-GFP
BcTol1^{K122R}-GFP	pNAN-OGG	BcTol1^{K122R}-GFP
BcTol1$^{\Delta VHS}$-GFP	pNAN-OGG	BcTol1$^{\Delta VHS}$-GFP
BcCcp1^{K101A}-Flag	phz126-olicP-Flag	BcCcp1^{K101A}-Flag
BcCcp1^{H131L}-Flag	phz126-olicP-Flag	BcCcp1^{H131L}-Flag
BcCcp1-5'-HPH-3'	N/A	ΔBcCcp1
BcCcp1-pBS-neo	pBS-neo	ΔBcCcp1-C
BcCcp1^{H131L}-pBS-neo	pBS-neo	BcCcp1^{H131L}
BcCcp1^{K101A}-pBS-neo	pBS-neo	BcCcp1^{K101A}
BcCcp1-mCherry	pNAB-OCT	BcCcp1-mCherry
BcRtt109-5'-HPH-3'	N/A	ΔBcRtt109
BcRtt109-Flag	phz126-olicP-Flag	OE-BcRtt109
BcRtt109-GFP	pNAN-OGG	BcRtt109-GFP
BcTol1-Flag	phz126-olicP-Flag	BcTol1-Flag
**Software and algorithms**		
ImageJ	National Institutes of Health	https://imagej.nih.gov/ij/
MEGA7	MEGA Software	https://www.megasoftware.net/
The SAS System for windows V8	SAS Institute	N/A

(*Continued on next page*)

Please cite this article in press as: Yang et al., Broad-spectrum chemicals block ROS detoxification to prevent plant fungal invasion, Current Biology (2022), https://doi.org/10.1016/j.cub.2022.07.022

CellPress
OPEN ACCESS

Continued

REAGENT or RESOURCE	SOURCE	IDENTIFIER
MODELLER 9.11	Sali[52]	N/A
Protein Preparation Wizard 2015	Sastry et al.[53]	https://www.schrodinger.com/Protein-Preparation-Wizard/
Excel 2010	Microsoft	https://products.offifice.com/en-us/excel
PowerPoint 2010	Microsoft	https://products.offifice.com/en-us/powerpoint
Other		
OpenSPRTM	Nicoya Lifesciences	N/A

RESOURCE AVAILABILITY

Lead contact

Further information and requests for resources and reagents should be directed to and will be fulfilled by the lead contact, Wenxing Liang (wliang1@qau.edu.cn).

Materials availability

Strains, antibodies and reagents used in this study will be made available upon request without any restriction.

Data and code availability

- All data reported in this paper will be shared by the lead contact upon request.
- This paper does not report original code.
- Any additional information required to reanalyze the data reported in this paper is available from the lead contact upon request.

EXPERIMENTAL MODEL AND SUBJECT DETAILS

The standard reference strain B05.10 of *B. cinerea* Pers. Fr. [*Botrytis fuckeliana* (de Bary) Whetzel] was isolated from *Vitis vinifera*.[54] All *B. cinerea* strains used in this study were grown on potato dextrose agar (PDA). Growth assays were conducted under 20 mM H_2O_2, and the percentage of mycelial radial growth inhibition (RGI) was measured after 3 days of incubation on PDA as previously described.[50]

METHOD DETAILS

Construction of gene deletion, complementation, site-directed mutagenesis, and GFP, Flag and mCherry fusion vectors

The gene deletion vectors were constructed using a double-joint PCR approach for each target gene.[55] The 5’ and 3’ flanking sequences of the target gene and hygromycin resistance gene cassette (HPH) were amplified with the primer pairs listed in Table S1. The resulting PCR products for each gene were transformed into B05.10 using protoplast formation and transformation of *B. cinerea*.[56]

To construct the complementation vector, the plasmid pBS-neo was used in this study.[50] The full-length target gene, including the promoter and terminator regions, was amplified from genomic DNA of the wild-type strain B05.10 and cloned between the NotI and SacI sites of pBS-neo to generate the complementation plasmid. Fusion PCR was employed to construct *B. cinerea* BcTol1^{K122Q}-pBS-neo and BcTol1^{K122R}-pBS-neo, and the resulting vectors were transformed into ΔBcTol1.[55] The primers used in this study are listed in Table S1. All the BcCcp1 mutants were constructed in a similar manner.

To construct the BcTol1-GFP/BcRtt109-GFP fusion cassette with its native promoter, the full-length target gene, including the promoter of BcTol1/BcRtt109, was amplified using the BcTol1-GFP-F/R or BcRtt109-GFP-F/R primers and assembled with NotI-digested pNAN-OGG[51] by the yeast gap repair approach. The BcCcp1-mCherry cassette was constructed using a similar strategy with NotI-digested pNAB-OCT.[51]

For site-directed mutagenesis of BcTol1, the BcTol1^{K122Q} or BcTol1^{K122R} gene with the native promoter region was generated by fusion PCR using the primers BcTol1-K122R -F/R or BcTol1-K122Q-F/R and cloned into the pNAN-OGG plasmid. Then, the constructs were transformed into protoplasts of ΔBcTol1 after sequencing. Site-directed mutants of BcCcp1 were constructed in pNAB-OCT using a similar method. Then, the constructs were transformed into protoplasts of ΔBcCcp1 after sequencing.

To overexpress the 3×Flag fusion proteins, namely, BcTol1-Flag, BcCcp1-Flag, BcRtt109-Flag, the enhanced olic promoter was amplified from the pNAN-OGG plasmid using the olic-F/R primers, cloned into the XhoI-digested phz126 vector to construct

phz126-olicP-Flag vector by the yeast gap repair approach. Then the target gene using the BcTol1-Flag-F/R, BcCcp1-Flag-F/R, or BcRtt109-Flag-F/R primers and cloned into the XhoI-digested phz126-olicP-Flag vector.

RNA extraction and quantitative reverse transcription PCR (qRT-PCR)

The expression levels of the target genes were tested by qRT-PCR using the $2^{-\Delta\Delta Ct}$ method.[57] Conidial suspensions (diluted in infection buffer: 6.7 mM KH_2PO_4, 6.7 mM glucose, 0.02% Tween-20; 10^6 conidia/ml) were dropped on mung bean leaves. After 4 days of incubation, the conidia and mycelia of the strains, including plant tissue, were harvested after 0, 6, 12, 24 and 36 h of incubation. RNA extraction, reverse transcription and qRT-PCR were performed using a protocol described previously.[50] RNA was extracted and reverse transcribed using All-In-One RT MasterMix (ABM). qPCR was performed using SYBR Premix Ex Taq (Takara). The actin gene was amplified as a reference. Three biological replicates were used for each sample.

Yeast two-hybrid assays

All the coding sequences of each target gene were amplified from the cDNA of B05.10 with the primer pairs listed in Table S1 and inserted into pGBKT7 and pGADT7 (Clontech). The resulting plasmids were cotransformed in pairs into *S. cerevisiae* strain Y2h-gold following the LiAc/SS-DNA/PEG transformation protocol.[58,59] The transformants were incubated at 30°C for 3 days on synthetic defined (SD) medium lacking Leu and Trp and then transferred to SD medium lacking His, Leu, Ade and Trp.

Co-immunoprecipitation (Co-IP) assays

The GFP, mCherry and 3×Flag fusion constructs were transformed in pairs or singly into B05.10 cells. Transformants expressing the fusion constructs were verified by PCR and Western blot assays. For Co-IP assays, mycelia of the strains were collected and ground in liquid nitrogen, and the powder was resuspended in lysis buffer (10 mM Tris-HCl, pH 7.5, 150 mM NaCl, 0.5 mM EDTA, 0.5% NP-40) with 2 mM PMSF and proteinase inhibitor cocktail (Roche). The supernatant lysates were then incubated with anti-GFP agarose (KT Health) at 4°C for 2 h with gently shaking. Finally, the resulted proteins eluted were detected with anti-Flag (Abcam) and anti-GFP antibodies.

Mass spectrometry analysis

Mycelia of the BcTol1-GFP and BcCcp1-mCherry carrying B05.10 strains were collected and ground in liquid nitrogen. The immunoprecipitation procedure was carried out as described in Co-IP assays. After IP, BcTol1-GFP or BcCcp1-mCherry pulled down was digested with trypsin and analyzed by mass spectrometry in PTM Biolabs (Hangzhou, China) as described.[60]

Generation of anti-K122ac-BcTol1 and anti-K101ubq-BcCcp1 antibodies

The antibody for BcTol1 K122 with site-specific acetylation was generated by using a BcTol1 acetylated peptide (FTRNIDAK(ac) FVQTVKC) conjugated to KLH as an antigen in rabbits by HUABIO (Hangzhou, China). The antibody for BcCcp1 K101 with site-specific ubiquitination was generated by using a BcCcp1 ubiquitinated peptide (KFDDYQK(ub)VYNEIA) conjugated to KLH as an antigen in rabbits by PTM Biolabs (Hangzhou, China). The specificity of the antibodies was tested by immunoblot analysis.

Western blot analysis

For detection of acetylated BcTol1, mycelia of all strains, namely, the BcTol1-GFP-expressing B05.10, ΔBcRtt109, and OE-BcRtt109 strains, were collected, ground in liquid nitrogen and resuspended in lysis buffer (10 mM Tris-HCl (pH 7.5), 150 mM NaCl, 0.5 mM EDTA, 0.5% NP-40) with 2 mM PMSF and proteinase inhibitor cocktail (Roche). The resulting supernatant was incubated with anti-GFP agarose (KT Health) at 4°C for 4 h with gentle shaking.[60] The eluted proteins were probed with an anti-GFP antibody (Abcam) and anti-K122ac-BcTol1 to detect the levels of BcTol1-GFP and its acetylation, respectively.

For secreted BcCcp1-mCherry detection, a total of 10^9 conidia were harvested from 10-day-old PDA cultures of WT::BcCcp1-mCherry, ΔBcTol1::BcCcp1-mCherry, $BcTol1^{K122Q}$::BcCcp1-mCherry, $BcTol1^{K122R}$::BcCcp1-mCherry, WT::$BcCcp1^{K101A}$-mCherry and WT::$BcCcp1^{H131L}$-mCherry. All conidia were incubated in 1/10 YEPD (0.2% peptone, 0.1% yeast extract, and 0.2% glucose) medium with tomato seedlings at 25°C for 6, 12, 24 and 36 h in a shaker. Then, the liquid cultures were harvested, and cold acetone was added at a final concentration of 80%. The mixtures were centrifuged at 12,000×g for 20 min at 4°C after incubation at -20°C overnight to separate the secreted protein. The total secreted proteins were dissolved in 1 × Gibco phosphate-buffered saline (PBS) and then boiled with protein loading buffer for 10 min. Then, the BcCcp1-mCherry level was determined with anti-mCherry antibody (Abcam).

Peroxidase activity measurement

The coding sequence of *BcCcp1* was amplified from the cDNA of *B. cinerea* and cloned into pET-28a(+). BcCcp1-His_6, $BcCcp1^{K101A}$-His_6 and $BcCcp1^{H131L}$-His_6 were expressed in *E. coli* BL21 (DE3) cells. Bacterially expressed recombinant BcCcp1-His_6 proteins was purified and diluted in PBS to a final concentration of 1, 2, 4, or 10 μg/ml. Twenty microliters of protein solution was added in a 100 μl reaction mixture [50 mM sodium acetate buffer (pH 5.0) and 20 mM ABTS (Sigma)]. Absorbance was evaluated at a 420 nm wavelength using a spectrophotometer after 5 min of incubation at 25°C.[16,32] The experiments were repeated three times.

Plant cultivation conditions

Tomato, tobacco and mung bean seedlings used for pathogenicity analysis were grown in a growth chamber at 25 °C with 75% relative humidity and a 16-h light /8-h darkness photoperiod. Seeds of the wheat were placed on wet pledges and grown in a growth chamber at 25 °C with a 16-h light /8-h darkness photoperiod and 100% humidity for 2-3 days. Seeds of the rice were first soaked in water at room temperature for 2 days, and then 37 °C for 1 day before sowed. The rice seedlings were grown in a growth chamber at 25 °C with 75% relative humidity and a 16-h light /8-h darkness photoperiod.

Pathogenicity and infection-related morphogenesis assays

The pathogenicity test of *B. cinerea* was performed with mung bean leaves with 10 μl of conidial suspension (diluted in infection buffer: 6.7 mM KH_2PO_4, 6.7 mM glucose, 0.02% Tween-20; 10^6 conidia/ml). After 4 days of incubation, the lesion diameters were measured. The experiments were repeated three times.

For the pathogenicity test of *M. oryzae*, conidia of Guy11 were collected with sterile distilled water containing 0.1% Tween-20 and adjusted to a concentration of 10^5 conidia/ml. Rice leaf segments were cut off from 2-week-old rice and placed on water-soaked paper in a tray. Ten microliters of conidial suspension was dropped on the leaf segments. To maintain high humidity, the trays were covered with plastic film and incubated at 25°C for 4 days.

For the pathogenicity test of *F. graminearum*, conidia of PH-1 were harvested and adjusted to a concentration of 10^5 conidia/ml and dropped on coleoptiles with the tip removed (3-day-old wheat seedlings), followed by incubation at 25°C and 95% humidity for three days before examination.

For the pathogenicity test of *F. oxysporum* f. sp. *lycopersici* strain 4287, 2-week-old tomato seedlings were used for root dip infection for 10 min in conidial suspension (10^6 conidia/ml). The infected plants were kept in a plant growth chamber at 25°C and 90% relative humidity for another 3 weeks before examination. The severity of disease symptoms was recorded and scored according to values ranging from 1 to 5 as described previously.[60]

For DAB staining, after the mung bean leaves were co-incubated with 10 μl of conidial suspension for 24 h, the leaves were immersed in a 1 mg/ml solution of DAB in buffer (pH = 3.8) and incubated at room temperature for 8 h in the dark. Then, the leaves were bleached with 95% ethanol until the samples became colorless.[61]

ROS detoxification of BcCcp1 in *Nicotiana benthamiana*

Bacterially expressed recombinant BcCcp1-His_6 or $BcCcp1^{H131L}$-His_6 was purified and diluted in PBS to a final concentration of 200 μg/ml, mixed with pTRV2-BAX, and co-infiltrated into *N. benthamiana* leaves. Cell death caused by co-infiltration of BAX and BcCcp1 was evaluated 3 days post injection, and DAB staining was performed at 36 hpi. To evaluate the function of BcCcp1 in biotrophic plant pathogen infection, 200 μg/ml BcCcp1-His_6 or $BcCcp1^{H131L}$-His_6 was infiltrated into *N. benthamiana* before *P. capsici* was inoculated.

Fluorescence microscopy

Conidia of *B. cinerea* expressing BcRtt109-GFP were harvested and inoculated at a concentration of 1×10^7 conidia/mL in YEPD medium at 25°C with shaking at 150 rpm for 1 day. The mycelia were collected, washed with PBS (pH 7.4) and stained with 1 μg/mL DAPI (Sigma) at room temperature in darkness for 5 min. Fluorescence microscopy was performed using an EVOS M5000 microscope (Invitrogen).

Subcellular fractionation analysis

The mycelia of BcRtt109-GFP were harvested and ground in liquid nitrogen. The nuclear and cytosolic proteins of BcRtt109-GFP were extracted using a Nuclear Protein Extraction Kit (Solarbio) according to the manufacturer's instructions. The resulting proteins were separated by SDS-PAGE and detected using anti-GFP (Abcam), anti-H3 (Abcam), and anti-tubulin (PTM Biolabs) antibodies.

Lysine acetylation reaction assay *in vitro*

The coding sequences of *BcTol1* and *BcRtt109* were amplified from cDNA and cloned in pET-28a and pGEX-4T-2, respectively. GST, BcRtt109-GST, and BcTol1-His_6 were expressed in *E. coli* BL21 (DE3) cells. Recombinant proteins were purified as described previously.[60] Ten micrograms of BcRtt109-GST and 10 μg of BcTol1-His_6 were incubated with the acetyl group donor acetyl-CoA (0.2 mM) in a buffer containing 50 mM Tris-HCl (pH 8.0), 10% glycerol, 1 mM dithiothreitol, and 1 mM sodium butyrate and incubated for 1 h at 37°C. Ten micrograms of GST and 10 μg of His-BcTol1 were also incubated as a negative control. Then, 4× SDS-PAGE loading buffer was added, and the mixture was boiled for 10 min to stop the reaction. The resulting proteins were separated by SDS-PAGE and analyzed by Western blotting using anti-His_6 (Beyotime) and anti-K122ac antibodies.

Molecular docking analysis

The 3D structure of BcVHS was constructed by MODELLER 9.11.[52] The human STAM1 VHS domain (PDB: 3LDZ)[62] was selected as a template for homology modeling (protein sequence similarity = 36.30%). Preparation of the free protein structure was carried out using the *Protein Preparation Wizard*[53] (Schrödinger, LLC, New York, NY). The ChemDiv subdatabase was used for virtual screening and contained 46369 compounds. The compounds were prepared by the *LigPrep* module (Schrödinger, LLC, New York, NY), where the protonated states were predicted at pH 7.0 ± 2.0. The *Glide* module in Schrödinger was utilized to conduct the docking

procedure. A protein grid box with a size of 8×8×8 $Å^3$ was created by centering on residues K76, Y80 and K122 using the *Receptor grid generation* module. The cutoff value for the partial atomic charge was set to 0.15, and the scaling factor for van der Waals radii was set to 0.8. The screening was conducted through three different precision modes of *Glide*, including high-throughput virtual screening (HTVS), standard precision (SP) and extra precision (XP). The top 35%, 30% and 20% of compounds in each round of docking were taken into the next mode based on the ranking of the *Glide* scores. All the calculation parameters in this process came from the default setting.

Surface plasmon resonance (SPR) analysis

The coding sequences of BcTol1 were amplified from the cDNA of *B. cinerea* and cloned into pET-28a. The recombinant proteins were purified as described previously.[60] WT and mutant BcTol1-His_6 proteins were fixed on the NTA sensor chip by capture coupling. The 5664-0417 or 6623-1943 solution at different concentrations (1, 5, 10, 20, 50 nM) was diluted in running buffer (10 mM PBS (pH 7.4), 150 mM NaCl) with an equal volume of 1% DMSO and then injected sequentially into the chamber. The interaction of the recombinant proteins with the fixed 5664-0417 or 6623-1943 was detected by OpenSPRTM (Nicoya Lifesciences, Waterloo, Canada) at 25°C according to the instructions of the manufacturer. The flow rate was set to 20 μl/s, both the binding time and dissociation time were 250 s, and hydrochloric acid (pH 2.0) was used to regenerate the chips. A one-to-one diffusion-corrected model was fitted to the wavelength shifts corresponding to different drug concentrations. The data were retrieved and analyzed with TraceDrawer.

QUANTIFICATION AND STATISTICAL ANALYSIS

All data were analyzed by using a one-way ANOVA, with LSD's correction for multiple comparisons where appropriate. The presence of different letters above the mean values of three replicates indicates a significant difference between different samples ($p < 0.05$, ANOVA).

Current Biology, Volume *32*

Supplemental Information

Broad-spectrum chemicals block ROS detoxification to prevent plant fungal invasion

Qianqian Yang, Jinguang Yang, Yameng Wang, Juan Du, Jianan Zhang, Ben F. Luisi, and Wenxing Liang

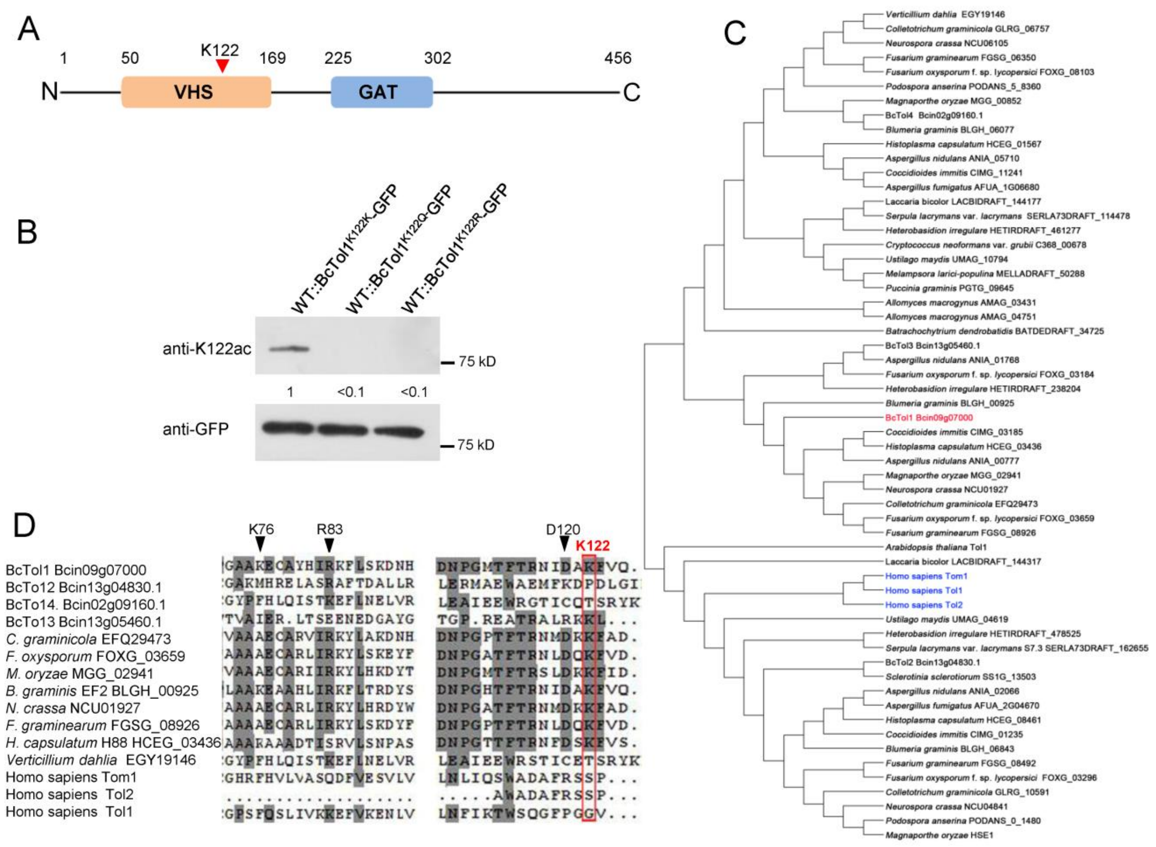

Figure S1. Acetylation of BcTol1 and phylogenetic analysis of its homologs. Related to Figure 1.

(A) The domain organization of BcTol1. The VHS and GAT domains, and the acetylated K122 residue were indicated.

(B) Acetylation of WT and K122 mutant BcTol1 proteins. BcTol1-GFP proteins pulled down from the indicated samples with GFP-Trap beads were probed with anti-K122ac and anti-GFP antibodies, respectively.

(C) Phylogenetic analysis of putative Tom1 and Tol proteins from *Homo sapiens*, *Arabidopsis thaliana* and 21 selected fungi. Maximum likelihood method was used to construct the phylogenetic tree.

(D) Alignment of the amino acid sequences of the VHS domain in putative Tom1 and Tol proteins from different species using DNAMAN6.0. K76, R83, D120 and K122 were indicated.

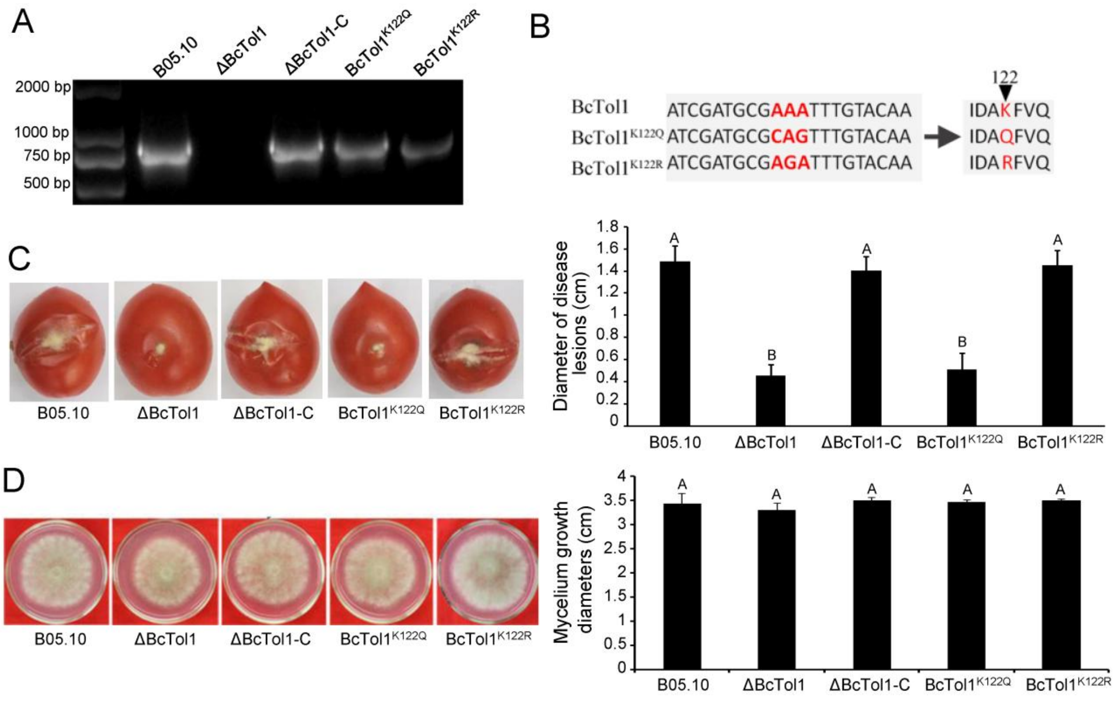

Figure S2. Generation of *BcTol1* mutant strains and phenotypic analysis. Related to Figure 1.

(A) PCR analysis to determine ΔBcTol1, ΔBcTol1-C, $BcTol1^{K122Q}$ and $BcTol1^{K122R}$ mutant strains. Genomic DNA was analyzed by PCR with the primer pairs indicated in Table S2.

(B) Sequence analysis showing mutation of K122 to Q or R.

(C) Virulence of B05.10 and BcTol1 mutant strains on tomato fruits. Photographs were taken 3 days after inoculation and the diameter of disease lesions was measured for 10 infected fruits.

(D) Morphology and mycelial diameter of B05.10 and BcTol1 mutant strains. Photographs were taken 60 h after incubation. The bars denote the standard errors of four replicates.

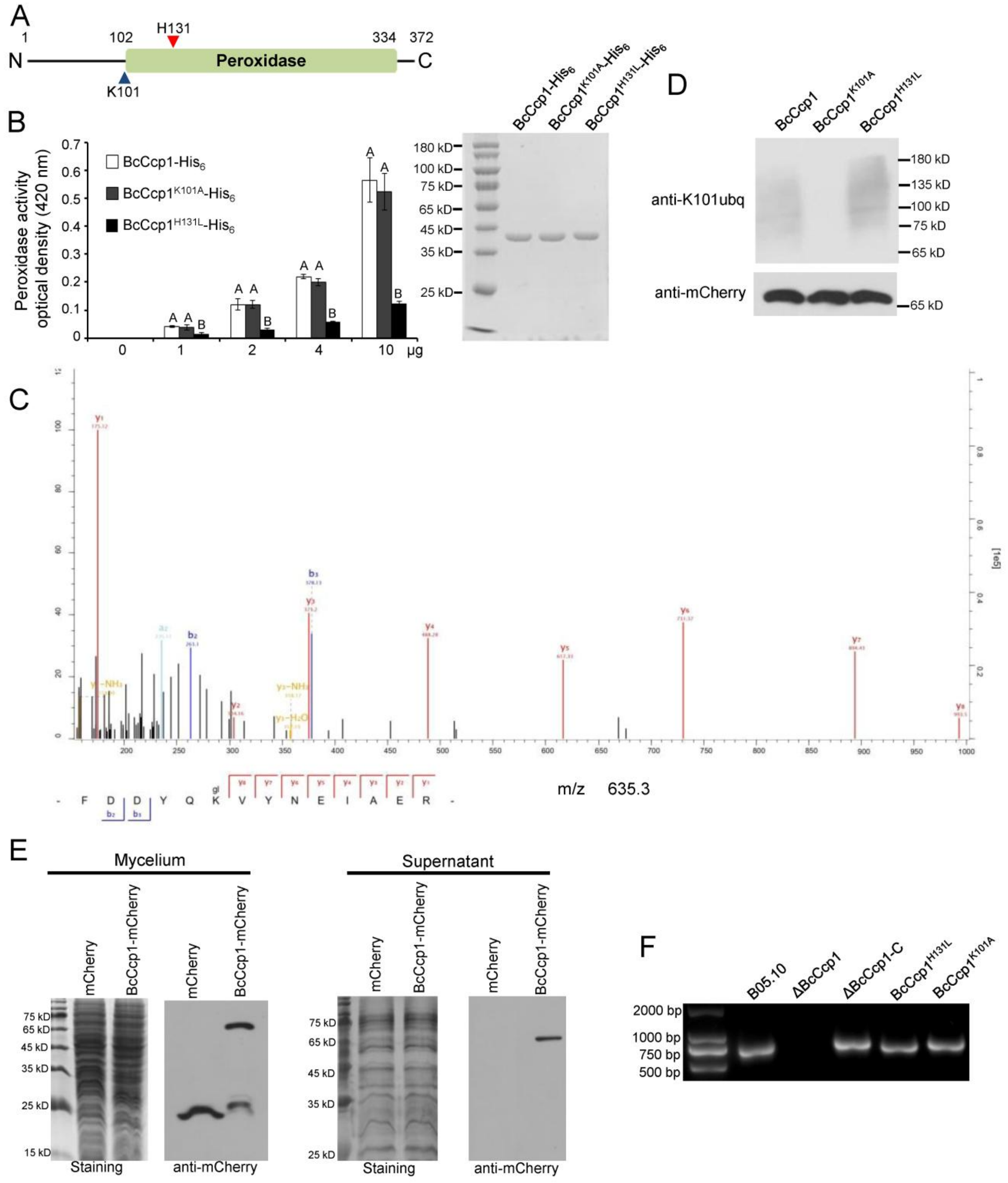

Figure S3. Activity, ubiquitination and secretion of BcCcp1, and generation of *BcCcp1* mutant strains. Related to Figures 2 and 3.

(A) The domain organization of BcCcp1. The peroxidase domain, the ubiquitinated K101 residue, and the core catalytic residue, H131, were indicated.

(B) Activity of WT and mutant BcCcp1 proteins. Peroxidase activity was determined by the ABTS oxidation assay with the purified BcCcp1-His$_6$ proteins. A coomassie brilliant blue (CBB) stained SDS-PAGE gel shows the purity of the WT and mutant BcCcp1 proteins.

(C) Annotation of representative tandem mass spectra from trypsin-digested BcCcp1-mCherry in *B. cinerea* depicting K101 ubiquitination.

(D) Ubiquitination of WT and mutant BcCcp1 proteins. BcCcp1-mCherry proteins pulled down from the mycelial extract using anti-mCherry antibody agarose beads were probed by anti-K101ubq and anti-mCherry antibodies, respectively.

(E) Secretion of BcCcp1. Conidia of the indicated strains were inoculated into 1/10 YEPD medium in the presence of two-week old tomato seedlings. Twenty-four hours after inoculation, total proteins extracted from the mycelia or culture supernatant were probed with anti-mCherry antibody. CBB or silver staining shows protein loading to each lane.

(F) PCR analysis to determine ΔBcCcp1, ΔBcCcp1-C, BcCcp1^{K122Q} and BcCcp1^{K122R} mutant strains. Genomic DNA was analyzed by PCR with the primer pairs indicated in Table S2.

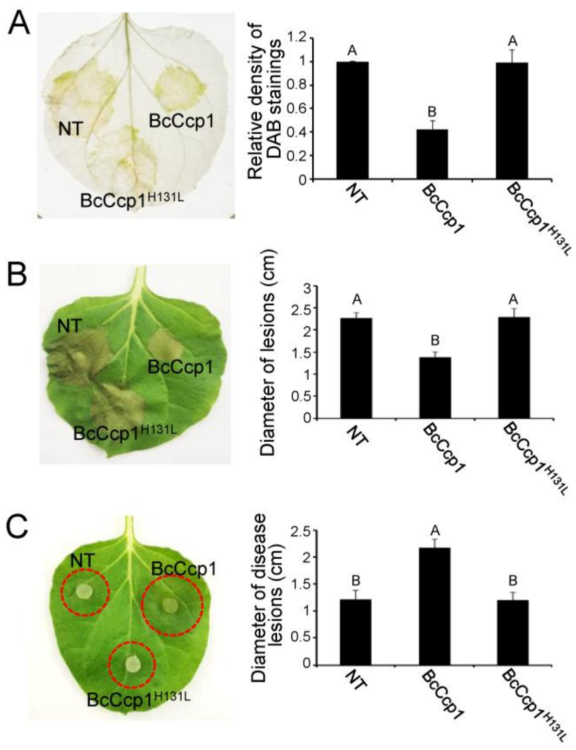

Figure S4. Effect of BcCcp1 on BAX caused H_2O_2 accumulation and cell death, and the infection of *Phytophthora capsici* in tobacco leaves. Related to Figures 3.

(A) Effect of BcCcp1 on H_2O_2 accumulation. Recombinant BcCcp1-His_6 or BcCcp1^{H131L}-His_6 was infiltrated into *N. benthamiana* leaves together with pTRV2-BAX containing *A. tumefaciens*, and DAB staining was performed at 36 hpi.

(B) Effect of BcCcp1 on cell death. Recombinant BcCcp1-His_6 or BcCcp1^{H131L}-His_6 was infiltrated into *N. benthamiana* leaves together with pTRV2-BAX containing *A. tumefaciens*. Photographs were taken 3 days after injection.

(C) Effect of BcCcp1 on *Phytophthora capsici* infection. Recombinant BcCcp1-His_6 or BcCcp1^{H131L}-His_6 was infiltrated into *N. benthamiana* leaves, and *P. capsici* was then inoculated. Photographs were taken 2 days after inoculation and diameter of disease lesions were measured. The presence of different letters above the mean values of three

replicates indicates a significant difference between different samples ($P < 0.05$, ANOVA). NT means no infiltration.

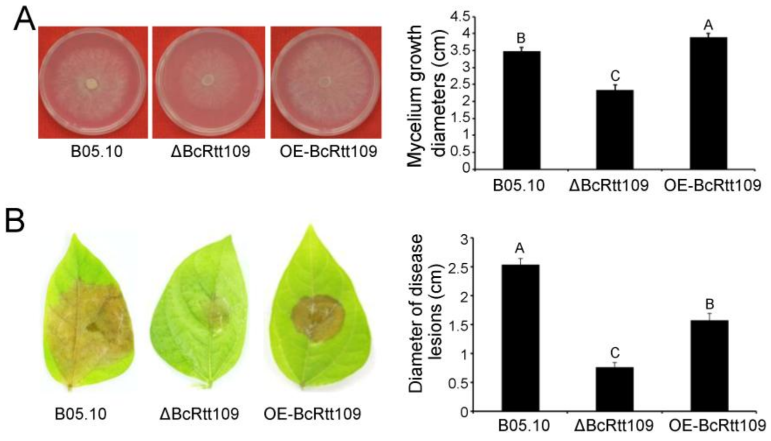

Figure S5. Phenotypic analysis of the ΔBcRtt109 and the OE-BcRtt109 strains. Related to Figures 4.

(A) Morphology and mycelial diameter of the B05.10, the ΔBcRtt109 and the OE-BcRtt109 strains. Photographs were taken 60 h after incubation. The bars denote the standard errors of three replicates.

(B) Virulence of the B05.10, the ΔBcRtt109 and the OE-BcRtt109 strains on mung bean leaves. Photographs were taken 4 days after inoculation and the diameter of disease lesions was measured for 10 infected leaves of each strain.

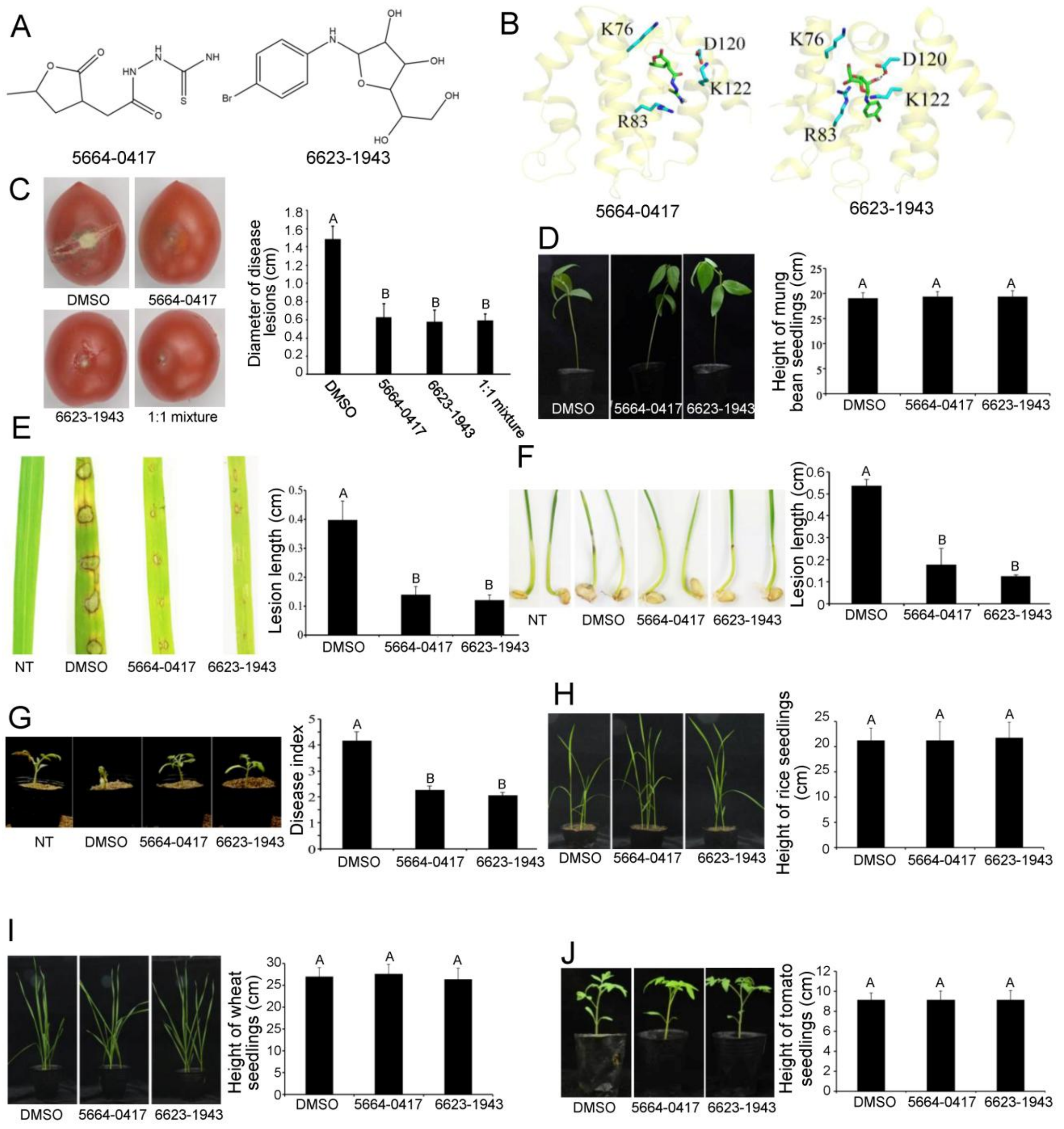

Figure S6. Effect of BcTol1 targeting chemicals on fungal pathogens and safety test. Related to Figures 5.

(A) Structure of 5664-0417 and 6623-1943.

(B) Re-docking showing the binding regions of 5664-0417 and 6623-1943 to BcTol1.

(C) Virulence of *B. cinerea* on tomato fruits with or without the application of 5664-0417, 6623-1943 or 1:1 mixture of the two chemicals.

(D) Safety test of the chemicals on mung bean seedlings.

(E) Virulence of *M. oryzae* on rice leaves with or without the application of 5664-0417 or 6623-1943. *M. oryzae* inoculation was performed simultaneously with treatment with 10 μM 5664-0417 or 6623-1943. Photographs were taken 5 days after inoculation and the length of disease lesions were measured.

(F) Virulence of *F. graminearum* on wheat coleoptiles with or without the application of 5664-0417 or 6623-1943. *F. graminearum* inoculation was performed simultaneously with treatment with 10 μM 5664-0417 or 6623-1943. Photographs were taken three days after inoculation and the diameter of disease lesions were measured.

(G) Virulence of *F. oxysporum* on tomato seedlings with or without the application of 5664-0417 or 6623-1943. *F. oxysporum* inoculation was performed simultaneously with treatment with 10 μM 5664-0417 or 6623-1943. Photographs were taken three weeks after inoculation and disease index was measured.

(H) Safety test of the chemicals on rice seedlings.

(I) Safety test of the chemicals on wheat seedlings.

(J) Safety test of the chemicals on tomato seedlings. For (B), (F)-(H), photographs were taken 2 weeks after spraying of 100 μM of the chemicals or DMSO and seedling heights were measured. The presence of different letters above the mean values of three replicates indicates a significant difference between different samples ($P < 0.05$, ANOVA).

Table S1. Primers used in the study. Related to STAR Methods.

Primer	Sequence(5'-3')	Relevant characteristics
BcTol1-up-F	GTTCAGGCTCTCTTGAAGAA	Amplify *BcTol1* upstream fragment for the construction of *BcTol1* deletion mutants
BcTol1-up-R	**GACCTCCACTAGCTCCAGCCAAGCC** ATATTCTTCATGGCT	
BcTol1-down-F	**ATAGAGTAGATGCCGACCGCGGGTT** ATCGTCAATGCTTCATCCC	Amplify *BcTol1* downstream fragment for the construction of *BcTol1* deletion mutants
BcTol1-down-R	CTGCTGATGCTGTGAGTTCA	
HPH-F	GGCTTGGCTGGAGCTAGTGGAGGTC	Amplify the hygromycin resistant gene *HPH*
HPH-R	AACCCGCGGTCGGCATCTACTCTAT	
BcTol1-K-F	ATACCGTGATCTTGAGGGGAA	Amplify the *BcTol1* deletion castle: up-HPH-down
BcTol1-K-R	ATGGAAGGTAAGGCTGCTAAG	
BcTol1-in-F	TTGAATTTGACTGACCATGGA	Identification of *BcTol1* deletion transformants
BcTol1-in-R	AGGGGGAAGGCGATGAGTA	
BcTol1-out-F	TGATTGCCACATGATGGCTT	Identification of *BcTol1* deletion transformants
BcTol1-out-R	TATCTGACACCGCAACACAA	
BcTol1-C-F	agaagcttAGGATGAGGCGTGTTTGTTT	Amplify full *BcTol1* including its up and dowm fragment for complement of the *BcTol1* deletion mutant
BcTol1-C-R	atgaattcGCATCGTCTTCACTGTTCTTG	
BcTol1-K122R-F	AATATCGATGCGAGATTTGTACAAA	Introduce K122R to BcTol1
BcTol1- K122R-R	TTTGTACAAATCTCGCATCGATATT	
BcTol1- K122Q-F	AATATCGATGCGCAGTTTGTACAAA	Introduce K122Q to BcTol1
BcTol1- K122Q-R	TTTGTACAAACTGCGCATCGATATT	
BcTol1-VHS-F	AGACCCTCAGCACGATACACCCGAGCA AGAGAAAATGATG	Amplify *BcTol1* without VHS domain
BcTol1-VHS-R	CATCATTTTCTCTTGCTCGGGTGTATCG TGCTGAGGGTCT	
BcTol1-GFP-F	**TGCAGCTGTGGAGCCGCATTCCC** AGGATGAGGCGTGTTTGTTT	Amplify full length *BcTol1* including its promoter for pNAN-OGG vector construction
BcTol1-GFP-R	**TACTTACCTCACCCTTGGAAACCAT** GTATCGATAGACAGGTTGC	
olic-F	**CTATAGGGCGAATTGGGTACTCAAAT TGGTT** TGCAGCTGTGGAGCCGCATT	Amplify full length olic promoter from pNAN-OGG for phz126-olicP-Flag vector construction
olic-R	**CTTTATAATCACCGTCATGGTCTTTG TAGTC** CTCGAGTTGGATCGATTGTGATGTGATG G	
BcTol1-Flag-F	**CCATCACATCACAATCGATCCAACC** ATGAAAGCCATGAAGAATATGG	Amplify full length BcTol1 for phz126-olicP-Flag vector construction
BcTol1- Flag -R	**CTTTATAATCACCGTCATGGTCTTTG TAGTC**GTATCGATAGACAGGTTGC	

BcActin-F	CATGGCTGGTCGTGATTTGA	*BcActin* for qPCR
BcActin-R	GAGGATTGACTGGCGGTTTG	
BcTol1-ex-F	CAGCACCGGACAATGATAC	*BcTol1* for qPCR
BcTol1-ex-R	CTGGTGGAGGAATCGGTG	
BcCcp1-ex-F	ATAAGGACGCTGCTGC	*BcCcp1* for qPCR
BcCcp1-ex-R	TTCCAGCTCCACTTCTCA	
BcRtt109-ex-F	ACTTGTTAGAAAGACCTGAG	*BcRtt10*9 for qPCR
BcRtt109-ex-R	TTGGTTCCTGTGACTAATTG	
BcTol1-BD-F	**GAGGACCTGCATATG**ATGAAAGCCA TGAAGAATATGGGCA	Amplify full length *BcTol1* for Y2H vector construction
BcTol1-BD-R	**CTCCATGGCCATATG**GTATCGATAGA CAGGTTGC	
Ubq-AD-F	**GTACCAGATTACGCTCATATG**ATGCA GATCTTCGTC	Amplify full length ubiquitin for Y2H vector construction
Ubq-AD-R	**ACTGGCCTCCATGGCCATATG**TTATT GACCACCACGAAG	
BcCcp1-AD-F	**GTACCAGATTACGCTCATATG**ATGGC ATCCGCTACTCG	Amplify full length *BcCcp1* for Y2H vector construction
BcCcp1-AD-R	**ACTGGCCTCCATGGCCATATG**CTAG GCAGTAGTAGGCTTGAATT	
Ubq- BcCcp1- F	TTCGTGGTGGTCAAATGGCATCCGCTA CTCG	Fusion of ubiquitin and BcCcp1
Ubq-BcCcp1- R	CGAGTAGCGGATGCCATTTGACCACCA CGAA	
BcCcp1-up-F	GCCAATGTGAAACGATGATTG	Amplify *BcCcp1* upstream fragment for the construction of *BcCcp1* deletion mutants
BcCcp1-up-R	**GACCTCCACTAGCTCCAGCCAAGCC** AGTAGCGGATGCCATTTTGA	
BcCcp1-down-F	**ATAGAGTAGATGCCGACCGCGGGTT** CTTTGGGGTGCAAAATTGGT	Amplify *BcCcp1* downstream fragment for the construction of *BcCcp1* deletion mutants
BcCcp1-down-R	ATGGTCCCTTCCCCTCAGAT	
BcCcp1-K-F	TCAAGTGGGTCGAGATTTGCT	Amplify the *BcCcp1* deletion castle: up-HPH-down
BcCcp1-K-R	ACATCACCCCTTACTTCCTCA	
BcCcp1-in-F	AATCAACACCTTTCAGATCC	Identification of *BcCcp1* deletion transformants
BcCcp1-in-R	CGTCCTTATCTTGTCTACCT	
BcCcp1-out-F	CAACCCATTTATCAAAATGGC	Identification of *BcCcp1* deletion transformants
BcCcp1-out-R	GACTTGGAGGTCTTGTCTTCG	
BcCcp1-C-F	**ATCGATAAGCTTGATATCGAATTC**GC CAATGTGAAACGATGATT	Amplify full *BcCcp1* including its up and down fragment for complement of the *BcCcp1* deletion mutant
BcCcp1-C-R	**TAGAACTAGTGGATCCCCCGGG**ATG GTCCCTTCCCCTCAGAT	
BcCcp1-mcherry-Na-F	**TGCAGCTGTGGAGCCGCATTCCC**GC CAATGTGAAACGATGATT	Amplify full length *BcCcp1* including its promoter for pNAB-OCT vector construction
BcCcp1-mcherry-R	**TACTTACCTCGCCCTTGCTTACCAT** GGCAGTAGTAGGCTTGAATTTCA	
BcCcp1-Flag-F	**CCATCACATCACAATCGATCCAACC** ATGGCATCCGCTACTCG	Amplify full length *BcCcp1* for phz126-olicP-Flag vector

BcCcp1- Flag -R	**CTTTATAATCACCGTCATGGTCTTTG TAGTC**GGCAGTAGTAGGCTTGAATTTCA	construction
BcCcp1-28a-F	**AGCAAATGGGTCGCGGATCCGAATTC** ATGGCATCCGCTACTCG	Amplify full length *BcCcp1* for Pet-28a vector construction
BcCcp1-28a-R	**TCAGTGGTGGTGGTGGTGGTGCTCG AGCTAGG** GGCAGTAGTAGGCTTGAATTTCA	
BcCcp1-K101A-F	GATTACCAAGCCGTTTAC	Introduce K101A to BcCcp1
BcCcp1-K101A-R	GTAAACGGCTTGGTAATC	
BcCcp1-H131L-F	TGGCGTGGTTGTGCAGC	Introduce H131L to BcCcp1
BcCcp1-H131L-R	GCTGCACAACCACGCCA	
BcRtt109-Flag-F	**CCATCACATCACAATCGATCCAACC**A TGGCTACTCGAGGGAGTTTTGG	Amplify full length *BcRtt109* for phz126-olicP-Flag vector construction
BcRtt109-Flag-R	**CTTTATAATCACCGTCATGGTCTTTG TAGTC**CAGGGCTGGCGCATTTGG	
BcRtt109-GFP-F	**TGCAGCTGTGGAGCCGCATTCCC**TA GCGTACAATGAAGTAGCT	Amplify full length *BcRtt109* including its promoter for pNAN-OGG vector construction
BcRtt109- GFP -R	**TACTTACCTCACCCTTGGAAACCAT**C AGGGCTGGCGCATTTGG	
BcRtt109-GST-F	TGGTTCCGCGTGGATCCATGGCTACTC GAGGGAGTTTTGG	Amplify full length *BcRtt109* for pGEX-4T-2 vector construction
BcRtt109-GST-R	GGAATTCCTGGGGATCCTTAGTTCTTTA CCTTTTTTCTAATCATACCAGC	
BcTol1-28a -F	**AGCAAATGGGTCGCGGATCCGAATT C**ATGAAAGCCATGAAGAATATGG	Amplify full length of *BcTol1* for Pet-28a vector construction
BcTol1-28a -R	**TCAGTGGTGGTGGTGGTGGTGCTCG AG**GTATCGATAGACAGGTTGCTTAC	
BcTol1-28aV-F	**GAAGGAGATATACCATGGCA**ATGAAA GCCATGAAGAATATGG	Amplify VHS domain of *BcTol1* for pET-28a vector construction
BcTol1-28aV-R	**GTGGTGGTGG TG CTCGAG**GTACGCT TGTACACTTCTGCT	
K76A-R83A-F	GGAGCTGCGGCCGAATGCGCCTACCAT ATTGCCAAGTTCCTC	Introduce K76A and R83A to BcTol1
K76A-R83A-R	GAGGAACTTGGCAATATGGTAGGCGCA TTCGGCCGCAGCTCC	
K122Q-D120A-F	AGGAATATCGCCGCGCAGTTTGTACAA	Introduce K122Q and D120A to BcTol1
K122Q-D120A-R	TTGTACAAACTGCGCGGCGATATTCCT	
